# Recent Developments in Carbon-Based Nanocomposites for Fuel Cell Applications: A Review

**DOI:** 10.3390/molecules27030761

**Published:** 2022-01-24

**Authors:** Tse-Wei Chen, Palraj Kalimuthu, Pitchaimani Veerakumar, King-Chuen Lin, Shen-Ming Chen, Rasu Ramachandran, Vinitha Mariyappan, Selvam Chitra

**Affiliations:** 1 Department of Materials, Imperial College London, London SW7 2AZ, UK; twchenchem@gmail.com; 2 School of Chemistry and Molecular Biosciences, University of Queensland, Brisbane 4072, Australia; p.kalimuthu@uq.edu.au; 3 Department of Chemistry, National Taiwan University, Taipei 10617, Taiwan; kclin@ntu.edu.tw; 4 Institute of Atomic and Molecular Sciences, Academia Sinica, Taipei 10617, Taiwan; 5 Electroanalysis and Bio-electrochemistry Laboratory, Department of Chemical Engineering and Biotechnology, National Taipei University of Technology, Taipei 106, Taiwan; vinithavicky80@gmail.com; 6 Department of Chemistry, The Madura College, Vidhya Nagar, T.P.K. Road, Madurai 625011, India; 7 Department of Chemistry, Alagappa Government Arts College, Karaikudi 630003, India; schitrachem@gmail.com

**Keywords:** carbon-based nanomaterials, oxygen reduction reaction, specific activity, durability, energy conversion, polarization curves

## Abstract

Carbon-based nanocomposites have developed as the most promising and emerging materials in nanoscience and technology during the last several years. They are microscopic materials that range in size from 1 to 100 nanometers. They may be distinguished from bulk materials by their size, shape, increased surface-to-volume ratio, and unique physical and chemical characteristics. Carbon nanocomposite matrixes are often created by combining more than two distinct solid phase types. The nanocomposites that were constructed exhibit unique properties, such as significantly enhanced toughness, mechanical strength, and thermal/electrochemical conductivity. As a result of these advantages, nanocomposites have been used in a variety of applications, including catalysts, electrochemical sensors, biosensors, and energy storage devices, among others. This study focuses on the usage of several forms of carbon nanomaterials, such as carbon aerogels, carbon nanofibers, graphene, carbon nanotubes, and fullerenes, in the development of hydrogen fuel cells. These fuel cells have been successfully employed in numerous commercial sectors in recent years, notably in the car industry, due to their cost-effectiveness, eco-friendliness, and long-cyclic durability. Further; we discuss the principles, reaction mechanisms, and cyclic stability of the fuel cells and also new strategies and future challenges related to the development of viable fuel cells.

## 1. Introduction 

In recent years, hydrocarbon-based (coal, gas, and oil) fossil fuels have predominantly been used to meet our global energy demand. However, fossil fuels can create severe damage to the environment by emitting toxic pollutants such as SO_x_, NO_x_, and CO_x_ [1,2]. Therefore, the development of clean and green energy storage technologies is essential to protect the environment and also fulfill the global energy demand [3]. There are numerous methods that have been used to make key efforts in the development of high-efficient-based energy conversion and storage technologies [4,5]. Among them, the water-splitting process is an important energy storage system that produces hydrogen efficiently in fuel cells, where electrocatalyst plays a crucial role [6]. In general, metal hydroxide-based electrode catalysts are used as electrode materials in fuel cells. However, they have several limitations, such as high cost, limited accessibility, and short service-life, restricting their contribution to meet energy storage technologies [7]. In order to reduce the cost of electrodes, researchers have been focused on developing carbon-supported electrocatalysts that are effectively used in several energy storage technologies, such as supercapacitors, fuel cells, solar cells, and batteries, due to their outstanding low-residual current, cost-effectiveness, and long-cyclic durability [8,9,10,11,12,13,14]. 

Fuels cells are classified as phosphoric acid fuel cell (PAFC), polymer electrolyte membrane fuel cell (PEMFC), alkaline fuel cell (AFC), molten carbonate fuel cell (MCFC), and solid-oxide fuel cell (SOFC) based on the types of electrolytes used [15]. Among them, PAFC, PEMFC, and AFC are appropriate for automobiles and portable applications because they run at low temperatures (300 °C), whereas MCFC and SOFC operate at relatively high temperatures (>500 °C) and may be suitable for stationary applications. Electrochemical performance, efficiency, and durability are crucial factors to consider when choosing electrode materials for fuel cell applications [16]. The overall electrochemical reaction process can occur at both anode and cathode as follows:

At anode, oxidation

(1) $$2H_{2}\;\to 4H^{+}+4e^{-}$$

At anode, oxidation

(2) $$O_{2}+4H^{+}+4e^{-}\to \;2H_{2}O$$

Overall reaction

(3) $$2H_{2}+O_{2}\;\to \;2H_{2}O$$

During the electrochemical reaction, electrons and protons are created at the anode (oxidation reaction), while water and heat are produced at the cathode (reduction reaction) [17,18]. 

Numerous methods have been used to develop carbon-supported nanocomposites, most notably sol–gel [19] microwave (MW) assisted [20], sonochemical [21], electrochemical [22], and hydrothermal (HT) [23]. Among them, the electrochemical technique is a promising one due to several merits, including shorter fabrication time, uniform and desired thickness of film deposition, and higher stability [24]. For instance, a multifunctional N and O co-doped 3D carbon aerogel has been prepared through the carbonization route. The fabricated carbon felt–polyvinyl alcohol–polyaniline CF–PVA–PANI catalyst showed excellent catalytic activity towards ORR activities [25]. Correspondingly, a robust bi-functional-based N-doped carbon aerogel modified nickel (Ni/NCA) composite was prepared using hydrazine hydrate as a reductant at high temperature. Interestingly, the developed Ni/NCA composite appeared as agglomerated metal particles, which exhibited better electrocatalytic activity and good cyclic durability for the electrocatalytic reduction of *p*-nitrophenol [26]. On the other hand, Lopez et al. [27] developed a novel type of N-doped carbon nanofiber by the electrospinning technique with an applied voltage of 15 kV, and it served as a remarkable catalyst for ORR application. Further, an effective electrodeposition approach was used to successfully construct a simple pathway for the fabrication of bimetallic supported fullerene-based (Pd-W@fullerene) composite with the applied potential ranges of 1.2 to 0.25 V. The constructed Pd-W@fullerene was shown to be a promising catalyst for energy applications [28]. In another report, a durable electrode was fabricated using mixed nanomaterials such as gold nanoparticles and multi-walled carbon nanotubes incorporated on graphene nanoribbon (Au–NT–G) by HT treatment and the resulting composite played a significant role in polymer electrolyte membrane fuel cell technology [29]. 

Here, we summarize a comprehensive analysis of synthetic strategies used to develop different types of carbon-based nanomaterials and their relevant fuel cell applications. Further, we investigate the important factors that induce catalytic performance of nanomaterials, such as size, shape, morphology, synergic effect, and chemical interactions. Moreover, we compared the stability and catalytic performance of carbon-based nanomaterials to the commercial catalyst. 

## 2. Carbon Aerogel-Based Electrode Materials 

Carbon aerogels (CAs) are considered as a promising electrode material for energy storage and conversion applications in fuel cells, supercapacitors, and batteries due to their inherent characteristics, including massive specific surface area (SSA), a large pore volume, excellent electrical conductivity, and outstanding chemical, mechanical, and thermal stabilities [30,31,32]. In addition, they can be prepared from cellulose biomass, which is one of the most naturally abundant and cost-effective biopolymers [33]. For instance, Yang et al. developed N-doped CAs (NCAs) based on pyrolysis of polyacrylonitrile at controlled temperatures ranging from 600 to 900 °C [34]. The resulting nanocomposite was used for ORR application in the microbial fuel cell (MFC). The MFC not only produces electricity but is also used to treat wastewater [35]. In the MFC, organic waste matter is oxidized into electrons and protons at the microorganism-modified anode, whereas oxygen is reduced to water at the cathode. The developed NCAs catalyst was utilized as cathode material in MFC and displayed an impressive catalytic response towards ORR in 0.1 mol L^−1^ NaOH. Further, the N-doping causes the catalytic active site generation to be induced, and subsequently it enhances the catalytic response of the catalyst. The XPS data revealed that the N content in NCAs varied from 8 to 24% depending upon the temperature used for catalyst preparation. The onset (*E*_onset_) potential was found to be +0.81, +0.82, +0.85, and +0.84 V vs. RHE for the catalysts fabricated at different temperatures of 600, 700, 800, and 900 °C, respectively. These results corroborate that the catalyst fabricated at 800 °C (NCA-800) represented a better catalyst compared to other ensuing high pyridinic-N and graphitic-N contents. Furthermore, a maximum power density of 1048 ± 47 mW m^−2^ was achieved at the NCA catalyst when applied in the MFC, and this value is comparable to the value observed with the commercial Pt/C catalyst (1051 ± 28 mW m^−2^). 

**Figure 1:**
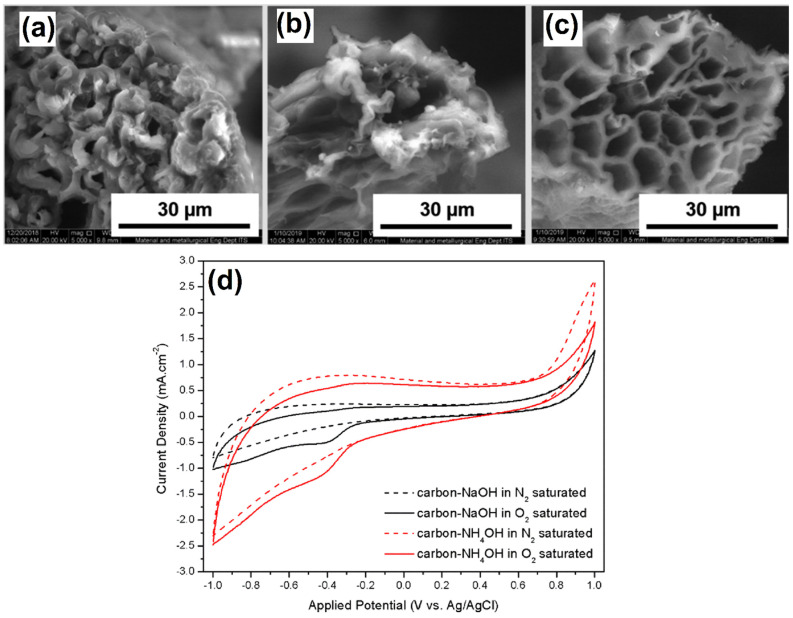
SEM images of the (**a**) cellulose aerogel, (**b**) carbon−NaOH aerogel, and (**c**) carbon−NH_4_OH aerogel. (**d**) CVs obtained for the different aerogels in O_2_ and N_2_ saturated solutions. Reprinted with permission from [36]. The American Chemical Society, Washington, DC, USA, 2020.

Porous NCAs were synthesized using two different routes, namely NH_4_OH–urea and NaOH–urea, by pyrolysis of cellulose aerogel derived from coir fibers [36]. Interestingly, these two routes rendered entirely different pore size CAs, as shown in Figure 1. The SEM image clearly indicates that the starting material (cellulose fibers) had a cylindrical shape with many internal pores. The estimated pores size and wall thickness were ~3 μm and ~3.2 μm, respectively (Figure 1a). In the NaOH–urea system, the internal structure of the aerogel disintegrated and obtained a sizeable internal pore with a broken wall (Figure 1b). On the other hand, the carbon aerogel prepared from NH_4_OH–urea system provided a stable structure with a larger internal pore size than the NaOH–urea system (Figure 1c). It revealed that ammonia is not only involved in the dissolution of cellulose, but it also creates defects in the carbon aerogel, and consequently, it causes an aerogel with a higher SSA and pore volume to be obtained. The internal pore size of the starting material, cellulose aerogels, was 200 μm, and it reduced to 150 and 176 μm upon pyrolysis of carbon−NaOH and carbon−NH_4_OH aerogels, respectively. Overall, the active SSA and pore volume increased from 70 to 3730 m^2^g^−1^ and 0.54 to 4.20 cm^3^ g^−1^, respectively. Further, the developed catalyst was evaluated in terms of ORR activity in an alkaline medium. Both carbon−NaOH and carbon−NH_4_OH aerogels displayed a well-pronounced reduction peak at −0.39 V (vs. Ag/AgCl) in the O_2_-saturated 0.1 mol L^−1^ KOH solution (Figure 1d). In contrast, no peak appeared in the N_2_-saturated 0.1 mol L^−1^ KOH solution, implying that the observed peak was associated with the ORR. Further, the carbon−NH_4_OH aerogel showed a current density of 1.05 mA cm^−2^, which was two times higher than that of the carbon−NaOH aerogel.

In another report, a robust macroporous carbon aerogel (MCA) was designed by pyrolysis of resorcinol–formaldehyde (RF) aerogel mixtures under an Ar atmosphere [37]. To achieve macropores in the composite, isocyanate derived polymer was introduced into the RF framework. It has been reported that the cross-linker isocyanate loses its chemical bonding with nanoparticles in the RF framework and develops the macropores. The developed macroporous aerogels were used as a substrate for the deposition of Pt. The coverage of Pt on aerogels was estimated to be 5.67 × 10^−11^ mol cm^−2^, which is equal to 57% of monolayer coverage. Finally, the as-prepared Pt coated aerogel was successfully used as an electrode material for fuel cell applications.

**Figure 2:**
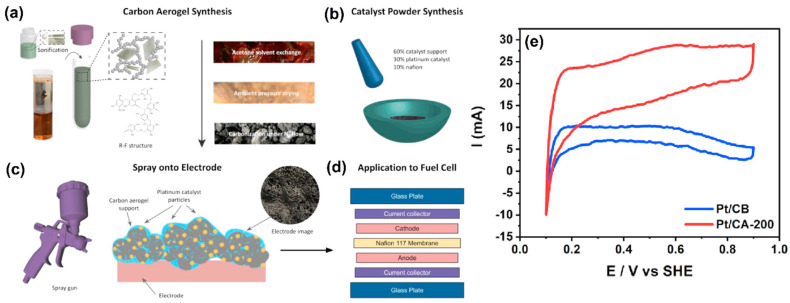
(**a**) Synthesis of CA, (**b**) Pt/catalyst (e.g., Pt/CA or Pt/CB) preparation, (**c**) application onto the electrode, and (**d**) membrane electrode assembly (MEA) preparation. (**e**) CVs of Pt/CB and Pt/CA-200 catalyst layer for ECSA analysis by hydrogen adsorption/desorption at a scan rate of 20 mV s^−1^. Reprinted with permission from [38]. Elsevier, Amsterdam, The Netherlands, 2021.

Recently, the development of porosity-tunable carbon aerogel for proton-exchange membrane fuel cells (PEMFCs) was demonstrated by Gu et al. [38]. It is well known that the diffusion of the reactants and products are directed to affect the performance of the PEMFCs. Therefore, it is essential to use the correct pore size of the membrane, depending on the analytes. The synthesis and electrode fabrication protocols are illustrated in Figure 2a–d. Firstly, the CA was synthesized by sol–gel polymerization of resorcinol and formaldehyde. Then, the CA was impregnated with carbon black (CB), and then platinum nanoparticles (PtNPs) were attached to the CA–CB mixture through chemical reduction by NaBH_4_. Further, a different ratio of R/Na_2_CO_3_ was used to prepare the various pore sizes of CAs. CA-100, CA-200, and CA-300 were obtained by mixing 1.54 g, 3.08 g, and 4.62 g of resorcinol with 0.016 g of Na_2_CO_3_. The pore size of CA played a crucial role in the deposition of PtNPs. It was revealed that the micropore structure (<2 nm) is not appropriate for deposition of 2–4 nm of PtNPs, whereas the mesoporous structure is highly suitable due to their bigger size that is around 2–50 nm. Further, the mesopore structure stimulates the ionomer loading and creates an effective network for adequate proton transportation for H_2_ and O_2_. Figure 2d shows the step-by-step construction of membrane electrode assembly, where the homogenous catalyst ink was airbrushed on the carbon paper and dried. Then, the catalyst coated carbon papers were hot-pressed with Nafion 117 membrane at 130 °C for 1 min. The developed materials were tested in the context of hydrogen adsorption/desorption reaction, and it was found that CA-200 exhibited better activity than other catalysts. The surface area was estimated for Pt/CA-200 and Pt/CB and found to be 188 m^2^ g^−^^1^ and 86.4 m^2^ g^−^^1^, respectively (Figure 2e). 

Another group [39] used the same precursors to synthesize CA for hydrogen storage applications. Na_2_CO_3_ is usually used to dry the wet catalyst. However, it causes shrinking of the catalyst. In order to avoid this issue, the authors used the organic condensing agent triethylamine (TEA). Different ratios of resorcinol and formaldehyde were used to develop the catalyst and characterize it using different techniques. The BET data revealed that the optimized catalyst (CA-1000) had a surface area of 545.03 m^2^ g^−1^, pore volume of 0.271 cm^3^ g^−1^, and pore size of 1.96 nm. Furthermore, the hydrogen storage efficiency was evaluated to be 4.0 wt%. 

Further, a highly conducting and mesoporous CA was developed using resorcinol and furfuraldehyde (FFA) as precursors for PEMFC [40]. The MW-assisted polyol process was employed to incorporate the PtNPs into the CA. TEM images of PtNPs-loaded CA (PtCA) display that PtNPs were uniformly distributed on the CA with an average particle size of 3.5 nm. The as-prepared catalyst was tested towards ORR, and the activity was compared to the commercial catalyst JM20; it was found that the catalyst exhibited an onset potential of 964 mV vs. RHE compared to 918 mV of the JM20. Additionally, the half-wave potential was estimated to be 814 mV, which was 27 mV positively shifted compared to the JM20. Further, the catalytic performance was evaluated towards PEMFCs, and it was found that the PtCA showed a power density of 536 mW cm^−^^2^ at 0.6 V, whereas JM20 exhibited a power density of 525 mW cm^−2^ under similar conditions.

Graphene (GR) is a 2D-material, and it has high electron conductivity, a large SSA, and outstanding chemical stability [41]. However, 2D-layered GR sheets readily undergo π−π restacking by van der Waals attraction, driving reduction of the surface area and consequently affecting the electronic properties of the materials [42]. To resolve this issue, Zhou et al. demonstrated the fabrication of 3D-graphene/carbon nanotube (GR–CNTs) aerogels and PVA acquired as an organic binder [43]. The CNTs were introduced into the GR to control the restacking of GR sheets. A solvothermal process was employed to incorporate the PtNPs onto GR–CNTs. The capacitance property of the developed catalyst was tested for use as electrode material in supercapacitors. It was found that Pt/GR–CNTs exhibited a relatively higher ECSA value (75.0 m^2^ g^−1^) than their counterparts, such as Pt/GR (30.0 m^2^ g^−1^) and Pt/CNTs (28.5 m^2^ g^−1^).

**Figure 3:**
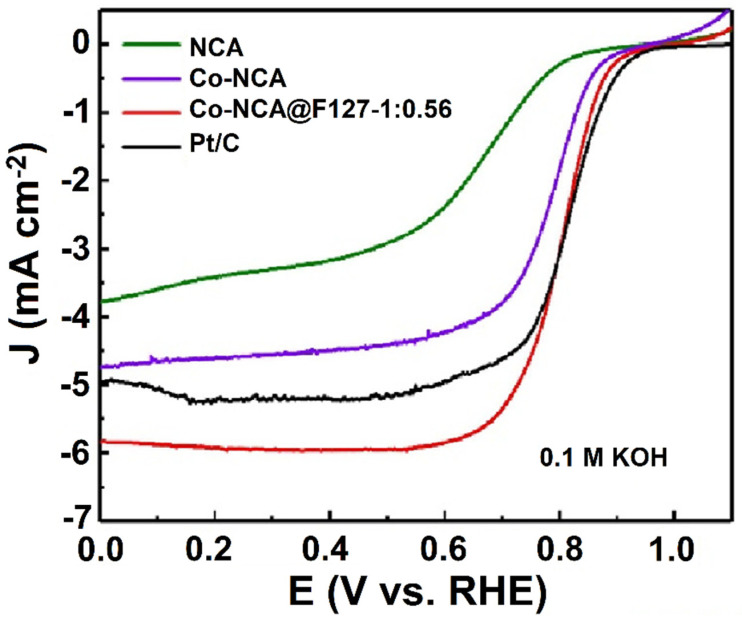
Rotating disc voltammetry for the ORR performance of NCA, Co-NCA, Co-NCA@F127-1, and Pt/C in O_2_-saturated 0.1 mol L^−1^ KOH solution at a scan rate of 10 mV s^−1^ and a rotation rate of 1600 rpm. Reprinted with permission from [45]. Elsevier, Amsterdam, The Netherlands, 2021.

The single atom-based transition-metal catalyst is considered an emerging nanomaterial for ORR applications. Several strategies were implemented to improve the catalytic performance of the single atom-based transition-metal catalyst, including electronic structure modulation, defect engineering, and integration with other suitable support [44]. The preparation of NCAs was demonstrated, where Co atoms (Co-NCA@F127-1) were atomically dispersed on chitosan template [45]. A polymeric material was obtained by reacting the *p*-aminophenol and F with HMTA under acidic and high-temperature conditions, and the resulting polymer was coated onto a chitosan template. To acquire the phenolic resin/Co^2+^ composite hydrogel, Co ions were chelated with the developed polymer that contained active functional groups, namely −OH, −NH_2_, and −COOH. Moreover, a surfactant, poloxamer (F127), was incorporated into the composite to improve the mechanical stability as well as control the morphology and pore structure of the CAs. The morphological characterization of CA by TEM revealed that it appeared as a 3D cross-linked coral structure. Further, ORR activity was investigated under alkaline conditions by the developed Co-NCA@F127-1, and the catalytic response was compared with the commercial Pt/C (Figure 3). It was found that the Co-NCA@F127-1 displayed an onset and half-wave potential of 0.935 V and 0.805 V vs. RHE, respectively, which is almost 128 mV higher than that of NCAs. In addition, in terms of diffusion limiting current density, the Co-NCA@F127-1 (5.96 mA cm^−2^) outperformed the commercial Pt/C (5.21 mA cm^−2^). 

**Figure 4:**
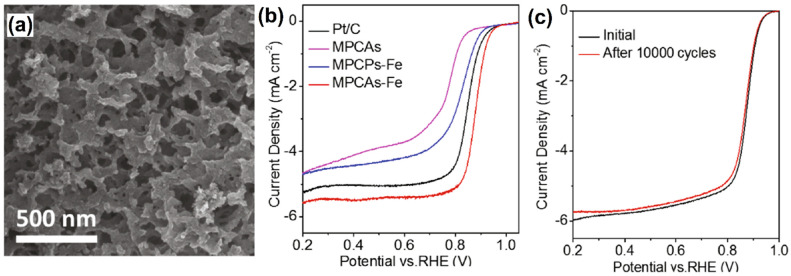
(**a**) SEM image of MPCAs–Fe and (**b**) ORR polarization curves of Pt/C, MPCAs, MPCAs–Fe, and MPCPs–Fe catalysts at a rotation rate of 1600 rpm in O_2_-saturated 0.1 mol L^−1^ KOH solution at a scan rate of 10 mV s^−1^. (**c**) ORR polarization curves of MPCAs–Fe before and after 10,000 CV cycles. Reprinted with permission from [46]. Elsevier, Amsterdam, The Netherlands, 2021.

A molecular-templating strategy was employed to develop microporous carbon aerogels (MPCAs) using a fast and straightforward polycondensation of 4,4′-biphenyl dicarboxaldehyde (BPDA) with octaaminophenyl polyhedral oligomeric silsesquioxane (POSS-NH_2_) [46]. The as-prepared MPCAs displayed a 3D interconnected macroporous structure along with a well-defined micropore framework (Figure 4a). To improve the catalytic efficiency of MPCAs, iron (III) nitrate was introduced with methylene blue by a simple annealing process. The obtained catalysts were investigated towards the ORR in 0.1 mol L^−1^ KOH, and it was found that the MPCAs–Fe^III^ exhibited remarkable catalytic response compared to the Fe-free MPCAs and the commercial Pt/C catalyst (Figure 4b). The Fe^III^ incorporated MPCAs showed half-wave potential and current density of 0.88 V and 5.8 mA cm^−2^, respectively, and these values were relatively superior to those of their counterparts, such as pristine MPCAs (0.76 V, 4.8 mA cm^−2^), Fe^III^ doped microporous carbon particles (MPCPs-Fe) (0.82 V, 4.8 mA cm^−2^), and also the commercial Pt/C catalyst (0.85 V, 5.3 mA cm^−2^). Further, the durability of the MPCAs–Fe^III^ catalyst was investigated by chronoamperometric measurements. Figure 4c depicts that there was a discernable change in the current response after 10,000 cycles, and it indicates that the catalyst has long-lasting catalytic activity for ORR. Moreover, no significant change in the morphology was observed for the catalyst after 10 h of continuous current measurement. These results revealed that MPCAs–Fe^III^ is a promising catalyst for fuel cell applications. 

## 3. Carbon Nanofiber-Based Electrode Materials 

Nanostructured carbons, including zero-dimensional (0D) carbon dots and quantum dots, one-dimensional (1D) carbon nanotubes (CNTs), and two-dimensional (2D) graphene, have attracted the interest of researchers in recent years [47]. Past research suggested that the size, structure, shape, and functionalization of carbon nanofibers are responsible for their characteristic applications in many fields, including materials science, nanotechnology, energy storage, biomedicine, tissue engineering, and environmental science [48,49,50,51,52]. It has been suggested that the high porosity formed by fiber interlacing allows the species to easily pass through the fiber layers without causing excessive pressure drop, which is advantageous for mass transfer [53]. In general, CNTs have received considerably greater attention than CNFs due to several advantages, such as lower levels of microstructural flaws, stronger tensile strength, smaller dimensions, and lower density. On the other hand, CNFs are a good alternative since their manufacturing technique is easily transferrable to industry. However, CNFs are approximately 2 to 3 times cheaper than SWCNTs or MWCNTs [54]. In particular, CNFs perform many purposes as active electrode materials in fuel cell research [55]. For instance, CNFs are effectively used in microbial fuel cells (MBFC) [56], direct methanol fuel cells (DMFC) [57], direct ethanol fuel cells (DEFC) [58], membrane fuel cells (MFC) [59], and polymer electrolyte membrane fuel cells (PEMFC) [60]. However, one disadvantage of CNFs is their limited surface area (less than 200 m^2^ g^−1^), which makes it difficult to disperse metal nanoparticles (MNPs) on CNF surfaces. Typically, very high metal concentrations are necessary in real fuel cell electrodes (20–60 wt%) [61,62]. The requirement of high metal concentration is due to the need to create a thin catalyst layer with a high density of catalytic sites, which is essential to reduce ohmic drop and mass transfer limits [63]. However, it is necessary to maintain a good dispersion of tiny MNPs as well as a robust resistance to corrosion phenomena for the catalyst. Electrochemical corrosion is highly connected to surface area and the presence of flaws in the graphitic structure. In this regard, it is critical to identify effective preparation processes capable of achieving good metal dispersion on low surface area supports as well as increasing the metallic phase’s resilience against sintering and dissolution [64,65]. We summarized the physical and electrochemical properties of carbon-based electrode materials used in fuel cell applications (Table 1).

**Figure 5:**
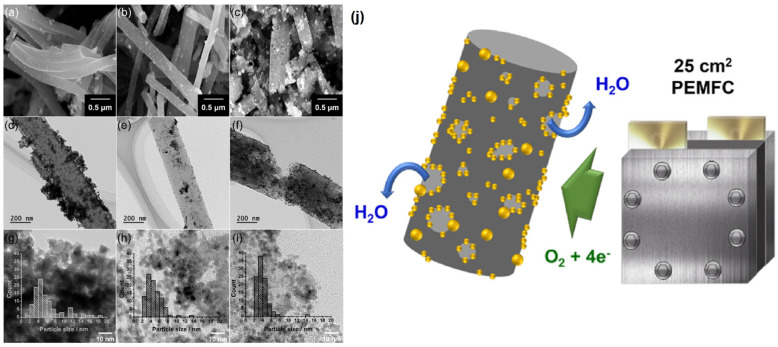
(**a**–**c**) FE-SEM, (**d**–**f**) TEM, and (**g**–**i**) HR-TEM images of Pt/CNF, Pt/GCF-HT, and Pt/GCF-(Co) (inset: Pt particle size distribution). (**j**) Scheme for the meso/macroporous graphitized carbon-supported Pt catalyst in the PEMFC cathode. Reprinted with permission from [74]. Elsevier, Amsterdam, The Netherlands, 2020.

Till now, the various forms such as porous [66], hollow [67], helical [68], twisted [69], and stacked [70] structures of CNF have been fabricated by various experimental approaches [71]. Furthermore, the functionalities of CNFs are easily tailored through both chemical and physical alterations, which is extremely beneficial for doping functional nanoscale building blocks on the surface of CNFs, which yield a variety of CNF-based functional nanomaterials. CNFs have been manufactured using a wide range of techniques, including catalytic chemical vapor deposition (CCVD) [72], arc discharge [73], and laser ablation [74]. While arc discharge and laser ablation produce high-purity CNFs, they require a large-scale operation and substantial production costs. Furthermore, because these technologies rely on carbon electrodes, they are inappropriate for large production in the long run. However, for many applications, a cost-effective approach to mass manufacturing is necessary. Pt catalysts on catalytically graphitized CNF performed exceptionally well in terms of ORR activity and durability [75]. Different forms of Pt catalysts such as Pt/CNF, Pt/GCF-HT, and Pt/GCF-(Co) were thoroughly investigated towards ORR. The additional heat treatment step on the catalytically graphitized CNF resulted in the formation of a unique pore structure with prominent meso/macropores. This may further enlarge the effective specific surface area and consequently provide more reactive sites. The morphologies of the Pt catalysts were investigated through a high-resolution FE-SEM, as shown in Figure 5a–c. Pt NPs were clearly loaded on fiber-based carbon supports with a thickness of 250 nm. A close examination of the morphology revealed that the Pt deposited as evenly for Pt/CNF and Pt/GCF-HT, whereas they developed porously on Pt/GCF-HT (Co). Figure 5d–f shows that Pt particles were aggregated on the CNF support, while uniformly dispersed on GCF-GCF-(Co). In comparison to Pt/CNF and Pt/GCF-HT, a reduced size of Pt NPs dispersed on GCF-(Co) according to HR-TEM images, as shown in Figure 5g–i. The average particle size was determined for Pt/CNF, Pt/GCF-HT, and Pt/GCF-(Co) and was found to be 6.0, 4.7, and 3.9 nm, respectively. In terms of PEMFC efficiency, the Pt/GCF-(Co)-HT attained a higher maximum power density of 0.85 W cm^−2^ in the active area of 25 cm^2^ upon a modest loading level of 0.1 mg Pt cm^−2^. When compared to the commercial Pt/C-TKK catalyst, the Pt/GCF-(Co)-HT catalyst had a superior mass transfer performance (over 2.2 A cm^2^). Further, Pt catalysts incorporated graphitized carbon in the PEMFC cathode; the 25 cm^2^ size of a single cell is schematically represented in Figure 5j [74].

**Figure 6:**
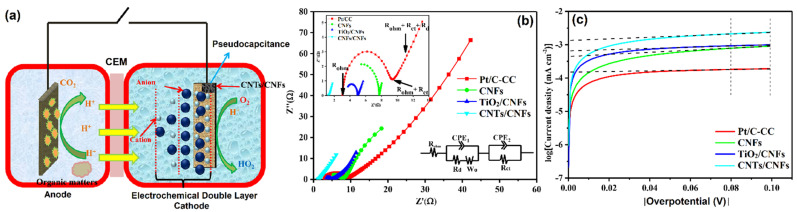
(**a**) The constructed new MFC, (**b**) Nyquist plots of EIS spectra, and (**c**) Tafel plots and the linear fitting of the exchange current at the overpotential between 80 mV and 100 mV. Reprinted with permission from [76]. Elsevier, Amsterdam, The Netherlands, 2019.

Cai and co-workers [76] developed a new MFC (Figure 6a) using CNTs/CNFs electrodes. The resulting catalyst had a high power density of 306 ± 14 mW·m^−2^, which was 140% superior to the conventional Pt/C. The longevity of the CNTs/CNFs catalyst was tested, and it was found that catalytic activity was retained up to 60 days without a voltage drop. The EIS was used to examine the internal resistance of the developed catalyst. The value of the first intersection of Nyquist plots and the Z’ axis was the value of an analogous electrical circuit consisting of an ohmic resistance (R_ohm_). The Nyquist plots were fitted with different vital experimental parameters such as double-layer capacitance, Warburg impedance, electrolyte diffusion resistance, pore adsorption capacitance, and charge-transfer resistance (Figure 6b). Tafel plots were used to compute exchange current density (i_0_), a crucial characteristic of ORR activity, by fitting the over potential from 80 to 100 mV (Figure 6c). Overall, the CNTs/CNFs electrode achieved a high apparent capacitance (0.68 ± 0.11 F·cm^−2^) and a long discharge time, which could be attributed to the simultaneous effects of electrochemical double-layer capacitance and pseudo-capacitance behavior. These characteristics indicated that CNTs/CNFs are promising catalysts for MFCs applications.

**Figure 7:**
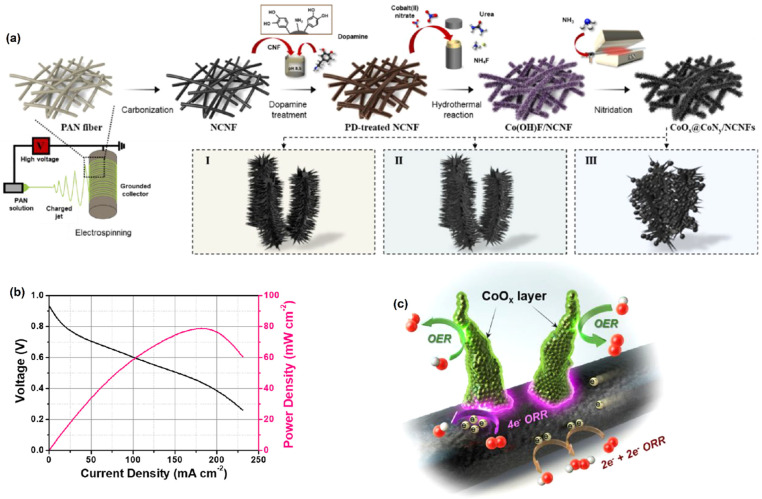
(**a**) Schematic illustration of the fabrication processes of CoO_x_@CoN_y_/NCNFs, (**b**) polarization curves of CoO_x_@CoN_y_/NCNF550-coated MEA in AMFC, and (**c**) proposed reaction scheme of CoO_x_@CoN_y_/NCNF toward ORR/OER. Reprinted with permission from [77]. The American Chemical Society, Washington, DC, USA, 2021.

Yoon et al. [77] demonstrated the CoO_x_@CoN_y_/NCNF550-catalyst-coated MEA used for ORR application. Figure 7a illustrates the fabrication processes of CoO_x_@CoNy/NCNFs achieved at various nitridation temperatures. Initially, 1D-N-doped CNF (NCNF) paper was made by electrospinning polyacrylonitrile (PAN) soaked in dimethyl formamide (DMF) solution and then carbonizing it. A consistent polydopamine (PD) coating layer formed on the surface of the NCNF paper by immersing it in a dopamine solution at ambient temperature. After 12 h of dopamine polymerization, the solution color changed from pale brown to dark brown. With a significant power density (~80 mW cm^−^^2^) of 177.2 mA cm^−^^2^, the CoOx@CoNy/NCNF550-catalyst-coated MEA demonstrated outstanding electrochemical ORR performance in AMFC (Figure 7b). It was revealed that the observed excellent ORR and OER activities were associated with the synergic effect between CoO_x_@CoN_y_ nanorods and NCNF. The ORR activity of NCNF was investigated, and the authors realized that it had a very poor catalytic response as a result of the 2e^−^ transfer process [78]. The ORR was assisted by the interfacial rim sites between the CoO_x_@CoN_y_ and NCNF, which allowed for moderate adsorption of intermediates and quick charge injection. Moreover, the Co_4_N nanorods’ oxidized coatings offered effective OER active sites with increased Co *d*-band vacancies. 

**Figure 8:**
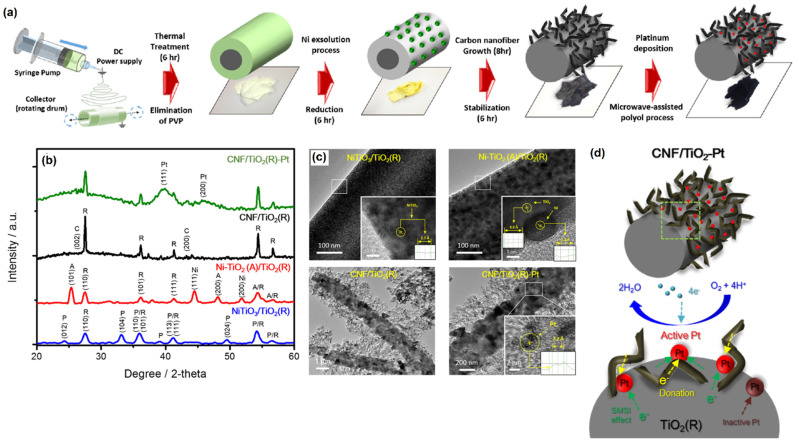
**(a)** Schematic illustration of synthesis procedure of the CNF/TiO_2_−Pt nanofibrous catalyst, (**b**) XRD patterns, (**c**) HR-TEM images of NiTiO_3_/TiO_2_, Ni-TiO_2_/TiO_2_, CNF/TiO_2_, and CNF/TiO_2_−Pt nanofibers, and (**d**) Schematic illustration of the Pt chemical state and catalytic reaction mechanism of the CNF/TiO_2_−Pt catalyst. Reprinted with permission from [79]. The American Chemical Society, Washington, DC, USA, 2018.

Jeon et al. [79] fabricated CNF/TiO_2_–Pt nanofibrous electrocatalyst via an in situ process through the protocol given in Figure 8a. The discovered nanofibrous catalyst had a larger active surface area and excellent ORR activity. The structural change that occurred upon the calcination and reduction procedure of the electrospun nanofibers can be noticed in the XRD analysis (Figure 8b). Furthermore, the HR-TEM photograph in Figure 8c depicts the whole synthesis flow of the nanofibrous composite structure, and it was discovered that before reduction, the surface appeared as a single phase of NiTiO_3_ with a typical interplanar distance of 2.7, harmonizing the (104) plane. On the other hand, after the reduction process, spherical Ni NPs with an average size of ~10 nm were well-deposited on the TiO_2_ nanofiber surface. The fabricated CNF/TiO_2_–Pt served as a better catalyst for ORR applications. Further, it was mentioned that the chemical structure of Pt NPs was modified on the CNF/TiO_2_–Pt surface due to the interaction between the Pt NPs and TiO_2_, as illustrated in Figure 8d.

## 4. Graphene-Based Electrode Materials 

Fuel cells are a type of energy conversion device that can generate electricity as long as fuel is available. Because the chemical energy of fuels is directly transformed into electricity, fuel cells have far better system efficiency than combustion engines, as well as lower pollution emissions. As a result, fuel cells are one of the most appealing technologies for addressing global energy and environmental concerns while also making our lives cleaner and more sustainable. An electrolyte is layered between two electrodes in an atypical fuel cell [80]. On the anode surface, the fuel is oxidized, and the liberated electrons pass through an external circuit to decrease O_2_ at the cathode. To complete the circuit, the mobile charge carriers (H^+^, OH^−^, CO_2_^3^^−^, or O^2−^) pass via electrolytes at the same time. Among various nanomaterials, GR and its derivatives have strong chemical, electrical, and mechanical capabilities, allowing them to be used as mass alternative materials in fuel cell applications. In recent years, many kinds of research have been focused on maximizing the potential usage of graphene-based materials in fuel cells. GR-based materials are excellent electrocatalyst supports because they increase the number of active sites and make electron transit easier for both fuel oxidation and ORR [81,82]. Due to their high electrocatalytic activity, high poisoning tolerance, and low cost, metal-free GR materials have been proved to be excellent candidates for ORR applications [83]. The impact of electronic structural change, doping configurations, defects, or graphene functional groups on the performance of fuel cells have been studied extensively [84]. The incorporation of graphene-based materials into polymer membranes can improve ionic conductivity and correspondingly reduce the fuel crossover [85]. High proton conductivity and impermeability to water, H_2_, and methanol make polymer membranes incorporating GR materials intriguing as fillers/additives [86]. In addition to electrolytes and electrodes, GR-based materials can increase current collection, fuel/air distribution, and bipolar plate stability [87].

GR is a one-atom-thick layer containing hexagonally arranged *sp*^2^-hybridized carbons [88,89]. In 2010, Andre Geim and Konstantin Novoselov received the Nobel Prize in Physics for their significant contributions to the development of GR-based catalysts [90]. Since then, GR has become the fastest-growing sector of science, spurring enormous effort and achievement in this field. GR-based materials include graphene oxide (GO), reduced graphene oxide (rGO), heteroatom-doped graphene, functionalized GR, and three-dimensional (3D) graphene present with a wide range of physical and chemical characteristics, as summarized in Table 2 [91,92]. 

**Figure 9:**
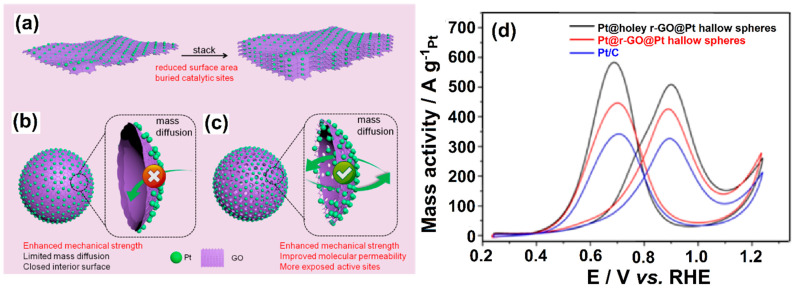
Schematic diagram of (**a**) 2D-GO/Pt NPs, (**b**) 3D-GO Pt@r-GO@Pt hollow nanospheres, and (**c**) 3D-Pt@holey r-GO@Pt hollow nanospheres, and (**d**) Pt mass-normalized CV curves recorded in an N_2_-saturated 0.5 mol L^−1^ of H_2_SO_4_ + CH_3_OH solution with a sweep rate of 50 mV s^−1^. Reprinted with permission from [95]. The American Chemical Society, Washington, DC, USA, 2018.

The use of GR materials in fuel cells has been shown to offer a number of advantages. For instance, GR-based materials act as potential ORR and fuel oxidation electrocatalysts due to their huge surface area and conductive properties [93]. Further, strong ionic conductivity, high tensile strength, and restricted fuel permeability are achieved while combining polymer membranes with GR [94]. Bipolar plate conductivity and corrosion resistance can be improved upon incorporating GR with an electrode material. Qiu et al. [95] used SiO_2_ nanosphere templates to create 3D holey rGO hollow nanospheres sandwiched by interior and exterior Pt nanoparticles (Pt@holeyr-GO@Pt hollow nanospheres) (Figure 9). The mass activity for MOR was 1.3- and 1.7-fold higher in Pt@holelyr-GO@Pt hollow nanospheres compared to Pt@r-GO@Pt hollow nanospheres and commercial Pt/C, respectively, due to increased mechanical strength and mass diffusion, as well as more exposed active sites. 

Modifying GR with heteroatom and functional groups provides more anchor sites for metal NPs, which significantly improves ethanol electrooxidation (EOR) activity and durability [96,97]. For instance, Yang et al. [96] used a diazo process to create aniline-functionalized graphene to bind Pd NPs for EOR. The mass activity of the catalyst with aniline groups (43.1 mA mg^−^^1^) was almost five times greater than that of the catalyst without aniline modification (8.9 mA mg^−^^1^) after 7200 s of testing, which was attributable to the well distributed Pd NPs and significant contact between aniline groups and Pd. The 3D-structured graphene can also improve ethanol transport by reducing catalyst aggregation and deactivation [98]. Yao et al. [99] used GO and ZIF-8 as precursors to spray-dry Pd encapsulated into hollow N-doped GR microspheres, displaying greater EOR activity (2490 mA mg^−^^1^) in alkaline medium than Pd/rGO (1232 mA mg^−^^1^). As previously stated, hollow N-doped GR microspheres are useful not only for immobilizing Pd NPs, but also for facilitating reactant diffusion for a better catalytic response [100].

## 5. Single-Walled Carbon Nanotube-Based Materials

**Figure 10:**
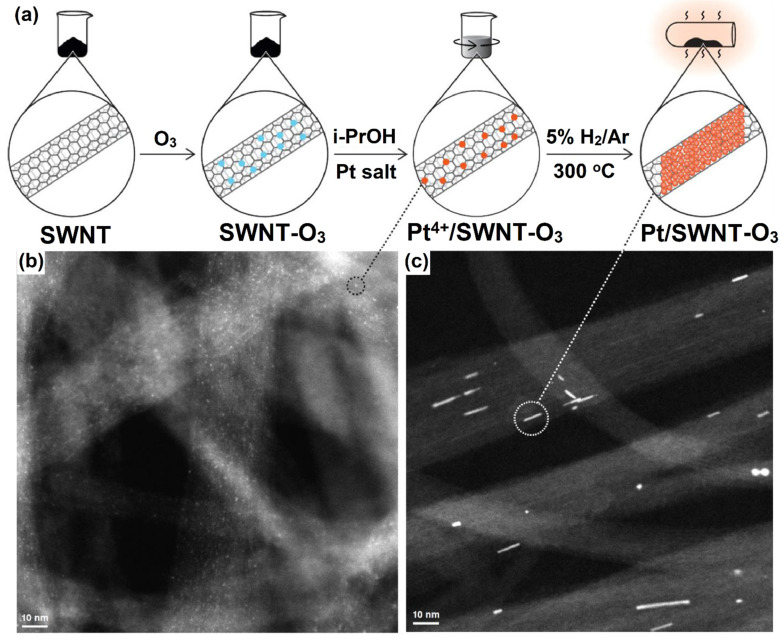
(**a**) Schematic illustration of the synthesis of Pt/SWNT-O_3_ with 3.9 wt% of Pt content; HAADF/STEM image of (**b**,**c**) CNT bundles with Pt/SWNT before and after heat treatment. Reprinted with permission from [108]. Elsevier, Amsterdam, The Netherlands, 2020.

Over the past three decades, the rapid development of nanotechnology occurred towards the 1D structural form of CNTs, which is considered the allotropic form of carbon [101]. SWCNTs have become the most versatile materials due to their outstanding properties such as unique structure, superior mechanical and electrical properties, excellent flexibility, low-processing cost, optical transparency, larger SSA, and excellent catalytic properties. Their diameters range from 0.7 to 10.0 nm [102]. So far, SWCNTs have attracted and hold great potential applications in various fields and have also expanded to solar cells [103], biomedicine [104], and energy storage devices [105], etc. The structural form of SWCNTs consists of a graphene sheet (single layer) rolled into a cylindrical tube format. In general, SWCNT-supported Pt-deposited thin film catalysts could facilitate better transport in DMFC applications [106]. Bimetallic based composites such as Pt-Ru/SWCNT and Pt-Mo/SWCNT were developed through a chemical reduction method, where the electrocatalysts were annealed for 2 h at 400 °C under N_2_ atmospheric conditions. Moreover, the as-synthesized Pt-Ru/SWCNT composite showed better current and power densities than Pt/SWCNTs catalysts [107]. Rajala and co-workers [108] fabricated PtNWs on SWCNTs (PtNWs/SWCNTs); the fabricated PtNWs/SWCNTs catalyst was pretreated with ozone, which renders polar surface groups on the SWCNT. The fabricated PtNWs/SWCNT-O_3_ composites were more hydrophilic in nature than non-ozonized compounds. This is due to an increase of oxygen-containing groups in the catalysts during the pretreatment with ozone. The synthesis procedure is outlined in Figure 10. Furthermore, the larger spherical agglomerates are more stable, and they do not create such NW structures. However, sub-nm Pt particles are required to produce PtNWs during the heat treatment, as shown in Figure 10b,c for before and after heat treatment of CNT bundles with PtNWs. Further, PtNWs/SWCNT-O_3_ catalysts outperformed in the hydrogen evolution reaction (HER) study with higher mass activity, which was estimated by DFT calculations [108]. 

**Figure 11:**
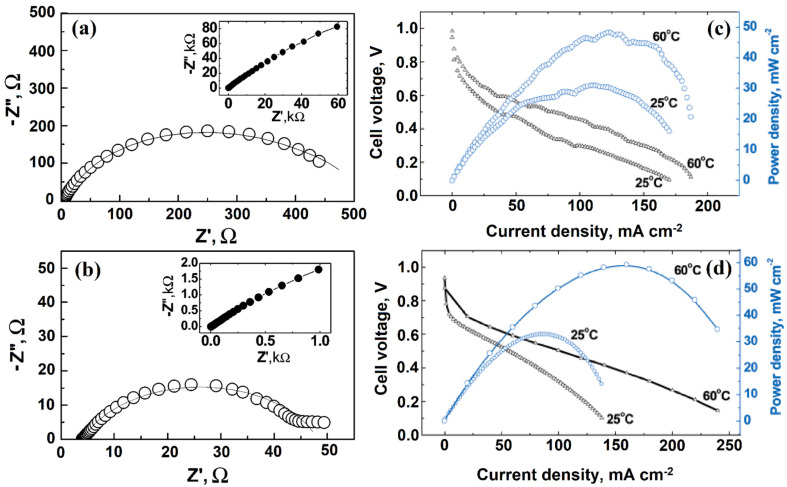
The AC impedance spectrum in Nyquist form for HER at an overpotential of 100 mV at the (**a**) CFE/CB/Pt electrode and (**b**) CFE/SWCNT/Pt electrode. (**c**) The power density and galvanostatic polarization data at different temperatures of an MEA prepared using a CFE/SWCNT/Pt anode and a CFE/CB/Pt cathode, and (**d**) the power density and galvanostatic polarization data at different temperatures of an MEA prepared using a CFE/SWCNT/Pt anode and cathode. Reprinted with permission from [112]. The American Chemical Society, Washington, DC, USA, 2005.

Fernandez et al. [109] tested the electrochemical behavior of SWCNT by using cyclic voltammetry and spectroscopic techniques. Moreover, SWCNTs were able to store hydrogen within their pores, which was confirmed through the galvanostatic charge–discharge method. Hu’s group [110] found that enclosing catalytically active potassium and iron metal nanoparticles in SWCNT catalysts improves ORR electrocatalytic activity. Further, Wu and Xu [111] showed that in comparison with the assembly using Pt supported on both MWCNT and SWCNT electrodes, during the electrochemical oxidation of methanol, Pt-SWCNT/NAF catalyst displayed significantly enhanced power density, lower onset potential, and lower R_CT_ values using CV and EIS analysis, whereas Pt-MWCNT/NAF catalysts displayed higher tolerance to CO poisoning and richness in oxygen-containing functional groups. A membrane electrode assembly (MEA) of SWCNT/CNF electrode was synthesized by Grishkumar et al. [112] via a simple electrophoretic technique. From the Nyquist plot, the SWCNT/Pt catalyst exhibited a lower R_CT_ value than that of CB/Pt (Figure 11a,b). The overall power output was investigated through galvanostatic polarization methods (Figure 11c,d). 

**Figure 12:**
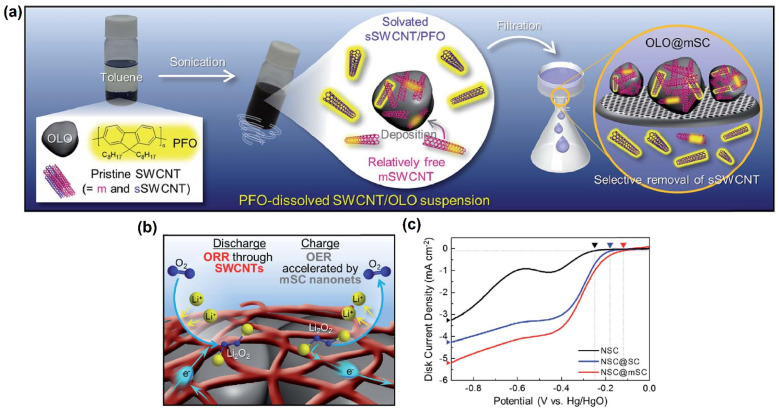
(**a**) Schematic illustration of the PFO-assisted one-pot surface engineering process for the preparation of OLO@m-SWCNT powders. (**b**) Conceptual illustration of the beneficial effect of the m-SWCNT nanonets on the bifunctional ORR/OER electrocatalytic activity (i.e., the ORR through the SWCNTs themselves and the OER accelerated by the m-SWCNT nanonets) of the NSC, and (**c**) ORR electrocatalytic activities of the pristine NSC, NSC@SWCNTs, and NSC@m-SWCNTs. Reproduced with permission [113]. Copyright, The Royal Society of Chemistry, London, UK, 2017.

Yoo et al. [113] prepared ivy-like conductive m-SWCNTs nanonets using a one-pot surface engineering strategy as displayed in Figure 12a. The authors employed Nd0.5Sr0.5CoO3 (NSC)-perovskite as a catalyst for the ORR/OER to test the feasibility of m-SWCNTs. Using a rotating ring-disk electrode (RRDE), the electrochemical ORR/OER performance of the NSC@m-SWCNTs was investigated in an oxygen-saturated 0.1 MKOH. The well-developed m-SWCNT nanonets are projected to increase electron conductivity on the NSC surface, making the NSC@m-SWCNTs electrochemical kinetics easier. The catalytic ORR activity of SWCNTs is widely recognized [114]. As a result, the NSC@m-SWCNTs can demonstrate bifunctional OER/ORR activities (see Figure 12b). The NSC@m-SWCNTs had a better ORR onset potential (−0.12 V against Hg/HgO) than the NSC (−0.25 V vs. Hg/HgO) and NSC@p-SWCNTs (−0.18 V vs. Hg/HgO) in the cathodic scan (Figure 12c). The NSC@m-SWCNTs’ greater catalytic activity was attributable to their higher diffusion-limiting current density than the NSC and NSC@p-SWCNTs. The NSC@m-SWCNTs are a potential bifunctional catalyst, according to these findings. Despite the fact that this outstanding study shows tremendous potential, there are few publications on the use of isolated SWCNTs as ORR/OER electrocatalysts. In another study, the two different types of carbon nanotube (SWCNT and SWCNT/PANI)-based electrocatalysts were developed by the arc discharge method. The electrocatalysts SSA of hydrophilic and hydrophobic values and their interactions were discussed. The as-prepared SWCNT/PANI catalysts showed significantly enhanced fuel cell efficiency [115].

## 6. Multi-Walled Carbon Nanotube-Based Electrode Materials

**Figure 13:**
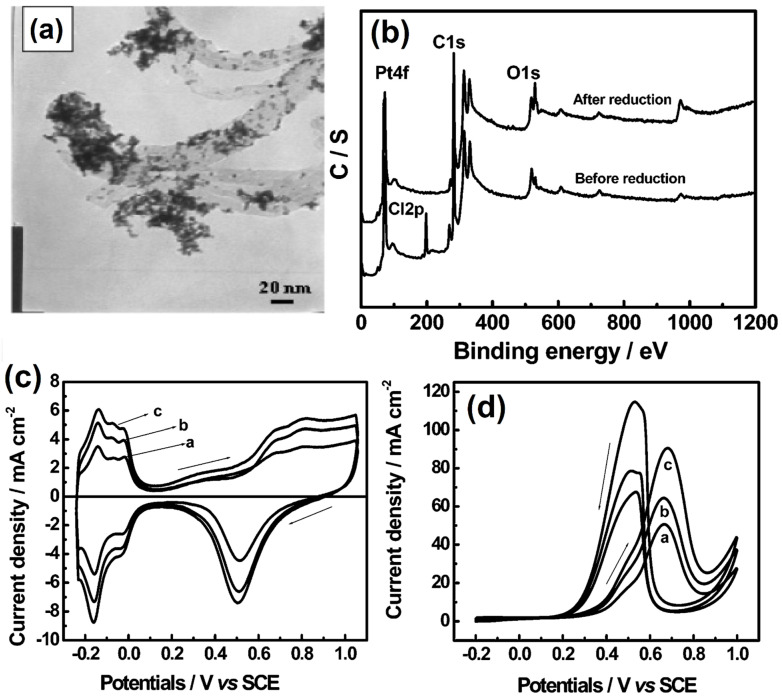
(**a**) TEM micrographs of Pt/MWCNT-10 nm, (**b**) XPS survey scan spectra of Pt/MWCNT nanocomposites before and after reduction treatment, and CVs of Pt/MWCNT nanocomposites prepared on MWCNTs with different diameters in (**c**) N_2_ saturated 0.5 mol L^−1^ H_2_SO_4_ and (**d**) CVs of Pt/MWCNT nanocomposites prepared on MWCNTs with different diameters in N_2_-saturated 0.5 mol L^−1^ H_2_SO_4_. Reprinted with permission from [129]. The American Chemical Society, Washington, DC, USA, 2006.

In recent years, MWCNTs-based electrode nanomaterials have attracted great attention due to their fascinating electrical, mechanical, thermal, and optical properties [116]. In 1991, Sumio Iijima developed carbon nanotubes from fullerene by the arc discharge method [117]. During the past three decades, carbon nanotube-based electrocatalysts have contributed to various electrochemical applications, such as electrochemical sensors [118], gas sensors [119], and energy storage devices [120,121,122]. MWCNTs are arranged in the form of cylindrical shapes made of *sp*^2^-carbon, and the diameter and length range from 3 to 30 nm and 40 to 50 nm, respectively [123]. Moreover, they consists of manifold-wrapped single graphene sheets into hollow tubes, whose outside diameters are 2 nm [124]. More specifically, MWCNTs are still the most promising candidate for developing next-generation transparent energy storage and conversion technology [125]. For the first time, Sun et al. [126] developed a porous polyaniline/multi-walled carbon nanotube-based Co_9_S_8_ (Co_9_S_8_+PPANI/MWCNT) composite that can act as a next-generation electrochemical hydrogen storage device and achieved a discharge capacity value of 689.2 mAh g^−1^. Interestingly, the spherical shaped nanoparticle of Pt_2_Ir/MWCNT composite has become a well-balanced bi-functional electrocatalyst, and more specifically a fresh active catalyst surface through electrode materials that play an important role in the development of direct methanol fuel cell applications. The catalyst has exhibited a mass activity value of 933.3 mA/mg_Pt_ [127]. Dogan et al. [128] introduced hexagonal-based boron nitrite with a conducting polymer supported MWCNT (h-BN-Ph-NH-CO-MWCNT) composite by coupling, acylation, and oxidation routes. The electrode materials have high van der Waals interactions with hydrogen, the acyl group playing an important role in enhancing their electrocatalytic activities. Moreover, the composite had an increasing hydrogen storage capacity under cryogenic conditions. Tian et al. [129] synthesized Pt nanoparticles supported on MWCNT (Pt/MWCNT) composite by the intermittent microwave irradiation (IMT) technique, and with H_2_PtCl_6_ used as a precursor. Figure 13a shows the TEM image of Pt/MWCNT; the Pt nanoparticles were uniformly placed on the MWCNT with a size range from 1.5 to 4.0 nm. The binding energy study of the Pt/MWCNT composite was carried out by XPS analysis before and after the reduction treatment process. Before the reduction process, the particles were clearly indicated at Pt 4f, Cl 2p, C 1s, and O 1s, respectively, whereas the Cl 2p peak vanished from the Pt/MWCNT composite after the reduction treatment method. The removal of halide ions can influence the catalytic activity of methanol fuel cells (Figure 13b). The electrochemical properties of Pt/MWCNT composite were studied using the cyclic voltammetric technique, using 0.5 M H_2_SO_4_ solution (Figure 13c). The as-synthesized Pt/MWCNT nanocomposite showed significantly enhanced methanol oxidation compared to E-TEK 40% Pt/C catalyst under acidic conditions (Figure 13d).

**Figure 14:**
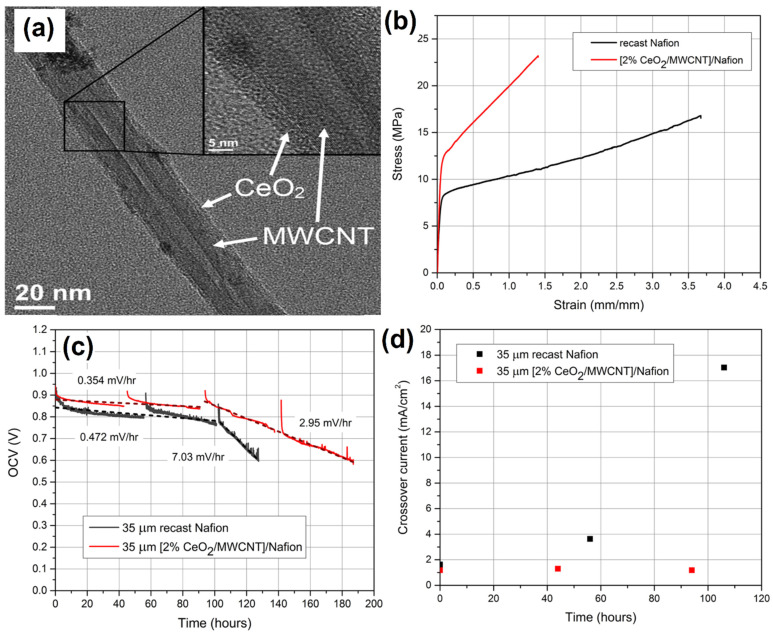
(**a**) TEM micrographs showing MWCNT after ceria treatment. (**b**) Typical stress–strain curves for NAF and [2% CeO_2_/MWCNT]/NAF membranes at 23 °C and 50% RH. (**c**) OCV decay of recast NAF and [2% CeO_2_/MWCNT]/NAF at 90 °C and 30% RH with H_2_/O_2_ flow rates of 100/200 sccm. (**d**) Gas crossover of recast NAF and [2% CeO_2_-MWCNT]/NAF membranes during OCV hold. Reprinted with permission from [130]. The American Chemical Society, Washington, DC, USA, 2014.

Generally, composite materials are often used for the development of polymer electrolyte membrane fuel cells. Barker et al. [130] showed the Nafion (NAF) proton exchange membrane and ceria-coated multi-walled carbon nanotube (CeO_2_/MWCNT/NAF) composite by wet chemical as well as solution-casting techniques. Figure 14a shows the TEM micrograph of the MWCNT wall after treatment of ceria nanoparticles, i.e., the diameter of the ceria particle was about 5 nm. The tensile strength and mechanical properties of CeO_2_/MWCNT/NAF composites were enhanced by the typical stress–strain study (Figure 14b). The electrode stability of the composite materials was tested using an open circuit potential value of the membrane degraded at 0.472 mV h^−1^, as shown in Figure 14c. The gas cross-over study revealed that the catalyst retained activity for up to 96 h (Figure 14d). 

**Figure 15:**
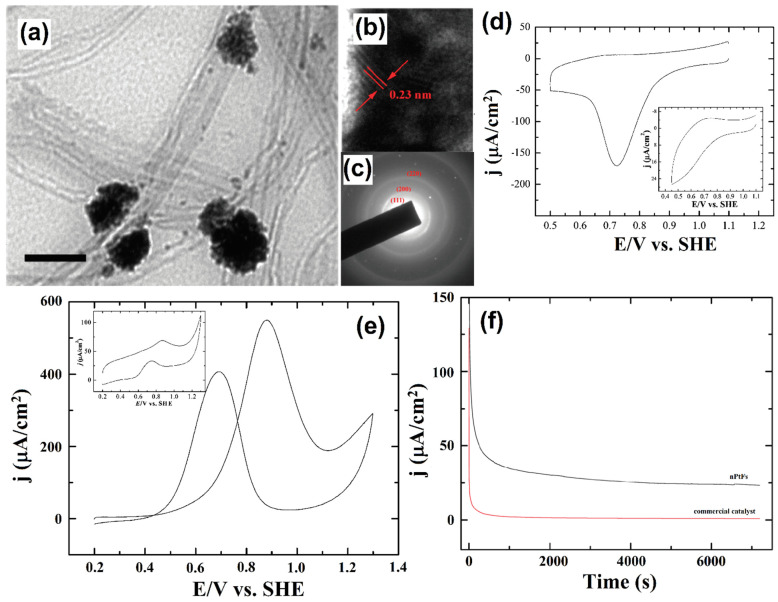
TEM (**a**,**b**) images and (**c**) SAED pattern obtained for the Pt nanoparticle decorated MWCNTs. The scale bar in (**b**) and (**c**) is 100 nm; (**d**) CV illustrates the electrocatalytic activity of nPtFs toward ORR in 0.5 mol L^−1^ H_2_SO_4_; scan rate: 25 mV s^−1^. (**e**) CV illustrating the electrocatalytic performance of nPtFs toward oxidation of methanol (0.1 mol L^−1^) in 0.5 mol L^−1^ H_2_SO_4_; scan rate: 25 mV s^−1^. Inset shows the voltammetric response of electrode modified with commercial catalyst toward methanol oxidation. (**f**) Chronoamperometric curves obtained for the oxidation of methanol (0.1 mol L^−1^) on nPtFs and commercial catalyst-modified electrodes. Reprinted with permission from [132]. The American Chemical Society, Washington, DC, USA, 2010.

Similarly, multi-walled carbon nanotube-based flower-like Pt nanostructure electrocatalysts were synthesized by the wet chemical reduction route. A TEM image of Pt-nanoparticle decorated MWCNT shows the flower and budlike morphologies and their average distribution of 80 nm (Figure 15a–c). The decorated Pt-MWCNT electrocatalysts were used to evaluate ORR by the CV technique in 0.5 M H_2_SO_4_ (Figure 15d). The electrocatalytic oxidation of methanol was evaluated by the CV technique, the anodic peak potentials were observed at ~0.9 V, and the methanol oxidation onset potential occurred at ~0.42 V (Figure 15e). The steady-state current was examined by using the chronopotentiometric technique (Figure 15f) [131]. Mink and co-workers successfully proposed a method to develop an MWCNT-based anode, and it was confirmed to be a feasible micro sized fuel cell device. The MWCNT-based anode materials hold promise as energy storage devices, and generated both electricity and water [132]. 

## 7. Fullerene-Based Electrode Materials 

**Figure 16:**
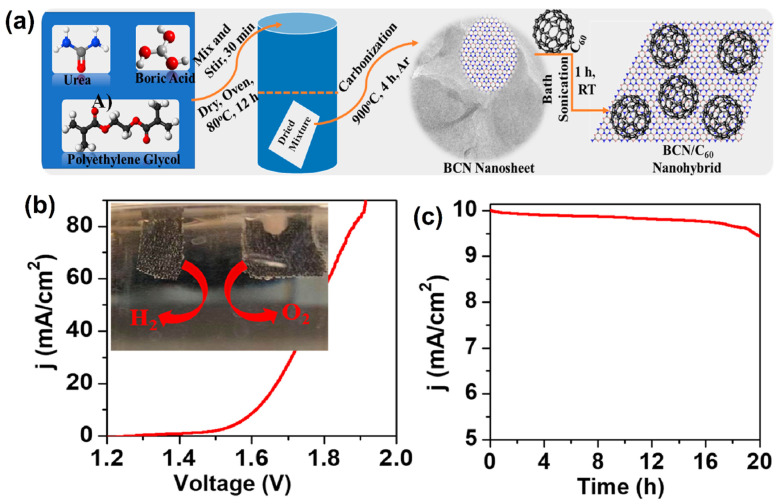
(**a**) Schematic representation of the synthesis of F/BCN nanohybrids, (**b**) LSV curve of the overall water splitting process using 10% F/BCN as both cathode and anode in a 0.5 mol L^−1^ NaOH solution. The inset in (**b**) shows the digital photograph for producing O_2_ (anode) and H_2_ (cathode) bubbles on the 10% F/BCN modified carbon cloth electrodes and (**c**) chronoamperometric measurements of 10% F/BCN for H_2_O electrolysis at 1.61 V for 20 h. Reprinted with permission from [144]. The American Chemical Society, Washington, DC, USA, 2021.

Buckminsterfullerene or C_60_ is an allotropic carbon present in different forms such as a spherical, tubular, and ellipsoid shape and was discovered by Kroto in 1985. It is represented as 0-D carbon-based materials [133]. Fullerene (C_60_) received considerable attention owing to its high electron transporting properties, good electron-accepting ability, and stable structural arrangements [134,135,136]. Moreover, C_60_-based electrode materials have been used as an efficient electrocatalyst in many fields, such as solar cells [137], batteries [138], biosensors [139], and fuel cells, etc. [140]. Having remarkable properties, such as convenience, low temperature operation, and high energy density of direct methanol fuel cells, using fullerene nanosheet modified (Pt/Ru/Sn/W fullerene) electrocatalysts could increase their catalytic activity with fuel cell efficiency [141]. The Pt-supported fullerene (Pt/C_60_(OH)_24-27_)-based electrocatalyst was synthesized through a simple process, where formic acid was used as a reducing agent. From TEM analysis, the agglomerated spherical-shaped fullerene (Pt/C_60_(OH)_24–27_) was obtained. Moreover, it can be used as an efficient electrocatalyst during the electrocatalytic oxidation of ethanol [142]. Rambabu and Bhat [143] developed a sulfonated polyether ether ketone supported sulfonated fullerene (SPEEK-Sfu)-based composite membrane by the diazotization reaction route. The SPEEK-Sfu composite membrane matrix showed better oxidation resistance with reduced methanol permeability in DMFCs. A new class of heterostructured boron nitride nanosheets was modified with fullerene molecules by a new strategic route to form 10% F/BCN multifunctional-based supramolecules (Figure 16a). Using the LSV technique, the constructed 10% F/BCN//10% F/BCN cell device exhibited remarkable current density (10 mA cm^−2^), and the insert digital image shows the overall water splitting process (Figure 16b). Finally, the 10% F/BCN electrocatalyst was tested through the chronoamperometric method, using a measurement period of about 20 h, showed good electrode stability (Figure 16c) [144]. 

Xiao et al. [145] reported an inorganic fullerene-based WS_2_ supported Pd nanoparticle catalyst by the sol-immobilization method, and the semi-spherical nature of the IF-Pd/WS_2_ catalyst exhibited excellent electrocatalytic activity towards HER with good cyclic stability. Generally, NAF can be used as an electrolyte for all types of electrode fabrication processes due to its good ionic conductivity and excellent electrode stability [146]. Rambabu et al. [147] prepared a composite membrane of NAF ionomer using functionalized fullerene (NAF^®^-FF) via the diazotization reaction method, where 4-benzenediazonium sulfonic acid was used as a precursor. Hence, the NAF^®^-FF composite membrane showed enhanced proton conductivity upon testing with different methanol concentration levels and exhibited a power density of 146 mW cm^−2^ in DMFC for NAF^®^-FF (1 wt%) with better stability. Zhang et al. [148] fabricated hybrid materials of graphene–fullerene, in addition to Pd NPs that were deposited on GO-PyrC_60_ via a simple HT approach. The hybrid-supported Pd/RGO-PyrC_60_ catalyst showed increased catalytic activity towards methanol oxidation. Feng et al. [149] designed a fullerene quantum dots (FQDs)-based CoNi layered double hydroxide (CoNi-LDH) nanosheet anchored with Ni foam (NF) via a self-assembled process. The decorated FQD/CoNi-LDH/NF catalyst showed excellent electrocatalytic activity for OER and HER as well as urea oxidation at ambient atmospheric conditions.

## 8. Conclusions and Perspectives

Carbon-based electrocatalysts have made major contributions in recent years to the development of cost-effective and environmentally-friendly hydrogen fuel cell energy storage technology that fulfills worldwide energy demand. It is apparent that contemporary research efforts are underway to develop low-cost carbon materials with improved power conversion efficiency, reduced CO_2_ and CO emissions, and greater durability. To build high-efficiency electrocatalysts, several techniques were adapted. Until now, much effort has gone into synthesizing carbon-based catalysts with various functionalization in order to obtain larger surface areas, uniform surface morphologies, and uniform nanoparticle dispersions, all of which can improve electrochemical properties and long-term durability of electrocatalysts. From these studies, a fundamental idea has been gained into the mechanistic details regarding the morphology, size effect, and synergic effect of the catalyst towards ORR and HER. Further, it has been demonstrated that the different forms of carbon-based composite electrodes, such as CAs, CNFs, fullerene, SWCNTs, MWCNTs, and GR, etc., were successfully used as promising candidates for fuel cell catalysts. Indeed, the ability to tailor the features of these interesting materials, especially their electrical characteristics, to reflect the specific requirements of each application promises to be a potent means of future improvements in this cutting-edge domain.

Most of the CNT-based electrocatalysts reviewed in the article showed a better catalytic response and were comparable to the expensive commercial catalysts. As a result, simple, low-cost, scalable, and controlled methods for producing CNT-based nanocomposites should always be designed, and various CNT-based applications are expected to move rapidly in the near future, opening the door to a plethora of new potentials in this promising and exciting field. Even though significant progress in developing low-cost CNT-based catalysts for hydrogen fuel cell applications has been made, several challenges remain to apply the developed catalysts for large-scale commercial applications. The major area of research is focus to improving the stability and long-term catalytic efficiency of the catalyst in alkaline and acidic media.

## Figures and Tables

**Table 1:** Summary of the various synthetic strategies, morphologies and their fuel cell properties of the recent reported carbon based catalysts.

Electrodes	Method	Morphology	SSA (m^2^ g^−1^)	Electrolyte (M)	Power Density (mW cm^−2^)	Ref.
N-CNF *^a^*	Pyrolysis	Nanofiber	916	0.1 KOH	10	[27]
NCAs *^b^*	Pyrolysis	Hollow structure	-	0.1 NaOH	1048 ± 47	[34]
Fe-Ricobendazole	Sacrificial route	Agglomerated particles	600	0.1 KCl:Buffer	0.200	[35]
CA *^c^*	Pyrolysis	Spherical structure	3730	0.1 KOH	1.05	[36]
Pt/CA	Sol-gel, NaBH_4_	Nanoparticles	700	−	333.4	[38]
Pt-MCA	Sol-gel	Mesoporous structure	613	0.5 H_2_SO_4_	536	[40]
Fe/Co/CNF	Electrospinning	Nanofiber	272	0.5 H_2_SO_4_	195	[48]
CNTs/CNFs	Electrospinning	Nanofiber	-	-	362 ± 20	[51]
GNF*^d^*-PtRu	Polyol	Nanofiber	16.9	-	19.2	[52]
WC/CNF*^e^*	Hydrothermal	Nanofiber	44.058	1 KOH	9.0	[58]

*^a^* Nitrogen-doped carbon nanofiber. *^b^* Nitrogen doped carbon aerogel. *^c^* Carbon aerogel. *^d^* Graphtized nano fiber. *^e^* Tungsten carbide based carbon nanofiber.

**Table 2:** The significant properties of Gr-based materials for fuel cell applications.

Gr	GO	Heteroatom Doped Gr	3D Gr
large surface area	strong hydrophilicity	tunable charge and spin density distribution	large surface area
high charge mobility	high proton conductivity	abundant active sites	high intrinsic electrical conductivity
high chemical stability	moderated electrical conductivity	high electrocatalytic activity	well-organized pore structure
strong mechanical strength	tunable electrochemical behavior	fast heterogeneous electron transfer rate	mechanical flexibility

## Data Availability

Not applicable.

## Abbreviations

AFC	Alkaline fuel cell
Au–NT–G	Gold nanoparticles–multi-walled carbon nanotube–graphene nanoribbon
BCN	Boron carbon nitride
BPDA	4,4′-biphenyl dicarboxaldehyde
CAs	Carbon aerogels
CB	Carbon black
CF–PVA–PANI	Carbon felt–polyvinyl alcohol–polyaniline
CFE	Carbon fiber electrode
CV	Cyclic voltammetry
DMFC	Direct methanol fuel cell
ECSA	Electrochemical surface area
EIS	Electrochemical impedance spectroscopy
EOR	Ethanol electro oxidation
FQDs	Fullerene quantum dots
FFA	Furfuraldehyde
GR	Graphene
HMTA	Hexamethylenetetramine
HER	Hydrogen evolution reaction
LSV	Linear sweep voltammetry
LDH	Layered double hydroxide
MFC	Microbial fuel cell
MCFC	Molten carbonate fuel cell
MEA	Membrane electrode assembly
MCA	Macroporous carbon aerogel
NAF	Nafion
Ni/NCA	Nitrogen doped carbon aerogel modified nickel
NF	Nickel foam
OCV	Open circuit voltage
ORR	Oxygen reduction reaction
PyrC_60_	Fullerene–pyrolidine
PAFC	Phosphoric acid fuel cell
PEMFCs	Proton-exchange membrane fuel cells
PtNPs	Platinum nanoparticles
PtNWs	Platinum nanowires
PVA	Polyvinyl alcohol
POSS-NH_2_	Octa-aminophenyl polyhedral oligomeric silsesquioxane
RF	Resorcinol–formaldehyde
R_CT_	Charge-transfer resistance
rGO	Reduced graphene oxide
Sfu	Sulfonated fullerene
SOFC	Solid-oxide fuel cell
SPEEK	Sulfonated polyether ether ketone
SSA	Specific surface area
SWCNT	Single-walled carbon nanotube
TEA	Triethylamine
TEM	Transmission electron microscopy

## References

[1] Abdelkareem M.A., Elsaid K., Wilberforce T., Kamil M., Sayed E.T., Olabi A. (2021). Environmental aspects of fuel cells; A review. Sci. Total Environ..

[2] Inumaru J., Hasegawa T., Shirai H., Nishida H., Noda N., Ohyama S. (2021). 1-Fossil Fuels Combustion and Environmental Issues. Advances in Power Boilers.

[3] Fan L., Tu Z., Chan S.H. (2021). Recent development of hydrogen and fuel cell technologies: A review. Energy Rep..

[4] Zhang H., Sun C. (2021). Cost-effective iron-based aqueous redox flow batteries for large-scale energy storage application: A review. J. Power Sources.

[5] Chai L., Hu Z., Wang X., Zhang L., Li T.-T., Hu Y., Pan J., Qian J., Huang S. (2020). Fe_7_C_3_ nanoparticles with in situ grown CNT on nitrogen doped hollow carbon cube with greatly enhanced conductivity and ORR performance for alkaline fuel cell. Carbon.

[6] Singla S., Sharma S., Basu S., Shetti N.P., Aminabhavi T.M. (2021). Photocatalytic water splitting hydrogen production via environmental begin carbon based nanomaterials. Int. J. Hydrogen Energy.

[7] Lu X.-F., Liao P.-Q., Wang J.-W., Wu J.-X., Chen X.-W., He C.-T., Zhang J.-P., Li G.-R., Chen X.-M. (2016). An Alkaline-Stable, Metal Hydroxide Mimicking Metal–Organic Framework for Efficient Electrocatalytic Oxygen Evolution. J. Am. Chem. Soc..

[8] Veerakumar P., Sangili A., Manavalan S., Thanasekaran P., Lin K.-C. (2020). Research progress on po-rous carbon supported metal/metal oxide nanomaterials for supercapacitor electrode applications. Ind. Eng. Chem. Res..

[9] Thangarasu S., Jung H.Y., Wee J.H., Kim Y.A., Roh S.H. (2021). A new strategy of carbon-Pd composite as a bipolar plate materials for unitized regenerative fuel cell system. Electrochim. Acta..

[10] Dong C., Xu B., Liu D., Moloney E.G., Tan F., Yue G., Liu R., Zhang D., Zhang W., Saidaminov M.I. (2021). Carbon-based an all-inorganic perovskite solar cells; progress, challenges and strategies towards 20% efficiency. Mater. Today.

[11] Liu H., Liu Q., Wang Y., Wang Y., Chou S., Hu Z., Zhang Z. (2021). Bifunctional carbon-based cathode catalysts for zinc-air battery: A review. Chin. Chem. Lett..

[12] Cheng D., Li Y., Zhang J., Tian M., Wang B., He Z., Dai L., Wang L. (2020). Recent advances in electro-spun carbon fiber electrode for vanadium redox flow battery; properties, structures and perspectives. Carbon.

[13] Vyas Y., Chundawat P., Dharmendra D., Punjabi P.B., Ameta C. (2021). Review on hydrogen production photo catalytically using carbon quantum dots; Future fuel. Int. J. Hydrogen Energy.

[14] Fonseca E.V., Yang W., Wang X., Rossi R., Logan B.E. (2021). Comparison of different chemical treat-ment of brush and flat carbon electrodes to improve performance of microbial fuel cells. Bioresour. Technol..

[15] Steele B.C.H., Heinzel A. (2001). Materials for fuel-cell technologies. Nature.

[16] Su H., Hu Y.H. (2020). Recent advances in graphene-based materials for fuel cell applications. Energy Sci. Eng..

[17] Peera S.G., Koutavarapu R., Akula S., Asokan A., Moni P., Selvaraj M., Balamurugan J., Kim S.O., Liu C., Sahu A.K. (2021). Carbon Nanofibers as Potential Catalyst Support for Fuel Cell Cathodes: A Review. Energy Fuels.

[18] (2019). Annual Energy Outlook, 2019 with Projects to 2050.

[19] Khanna S., Marathey P., Vanpariya A., Paneliya S., Mukhopadhyay I. (2021). In-situ preparation of ti-tania/graphene nanocomposite via a facile sol-gel strategy; A promising anodic materials for Li-ion batteries. Mater. Let..

[20] Mahalingam S., Ayyaru S., Aha Y.H. (2021). Facile one-pot microwave assisted synthesis of rGO-CuS-ZnS hybrid nanocomposite cathode catalysts for microbial fuel cell applications. Chemosphere.

[21] Choudhari A., Bhanvase B.A., Saharan V.K., Salame P., Hunge Y. (2020). Sonochemical preparation and characterization of rGO/SnO_2_ nanocomposite: Electrochemical and gas sensing performance. Ceram. Int..

[22] Feng X., Han G., Cai J., Wang X. (2022). Au@carbon quantum dots-Mxene nanocomposite as an electro-chemical sensor for sensitive detection of nitrate. J. Colloids Interface Sci..

[23] Gu Z., Zhang B., Asakura Y., Tsukuda S., Kato H., Kakihana M., Yin S. (2020). Alkali-assisted hydro-thermal preparation of g-C3N4/rGO nanocomposites with highly enhanced photocatalytic NOx removal ac-tivity. Appl. Surf. Sci..

[24] Li W.L.W., Tu M.T., Cao R., Fisher R.A. (2016). Metal-organic framework thin film; Electrochemical fabrication techniques and corresponding applications and perspectives. J. Mater. Chem. A.

[25] Kang Z., Wang Y., Yang C., Xu B., Wang L., Zhu Z. (2021). Multifunctional N and O co-doped 3D car-bon aerogel as a monolithic electrode for either enzyme immobilization, oxygen reduction and showing supercapacitance. Electrochim. Acta.

[26] Shanmugam P., Murthy A.P., Theerthagiri J., Wei W., Madhavan J., Kim H.S., Maiyalagan T., Xie J. (2019). Robust bifunctional catalytic activities of N-doped carbon aerogel-nickel composite for electrocatalytic hydrogen evolution and hydrogen of nitro compound. Int. J. Hydrogen Energy.

[27] Lopez R.O., Sanchez G.R., Schulz J.M.E., Lartundo L., Ortiz A.D. (2017). On site formation of N-doped carbon nanofibers, an efficient electroctalysts for fuel cell applications. Int. J. Hydrogen Energy.

[28] Bhavani K.S., Anusha T., Shanmikha J.V., Brahman P.K. (2021). Enhanced electrocatalytic activity of methanol and ethanol oxidation in alkaline medium at bimetallic nanoparticles electrochemically decorated fullerene-C60 nanocomposite electrocatalyst; An efficient anode materials for alcohol fuel cell applications. Electroanal.

[29] Latif H., Wasif D., Rasheed S., Sattar A., Rafique M.S., Anwar A.W., Zaheer S., Shabbir S.A., Imtiaz A., Qutab M. (2020). Gold nanoparticles mixed multiwall carbon nanotubes, supported on graphene nanoribbons (Au-NT-G) as an efficient reduction electrode for Polymer Electrolyte Membrane fuel cells (PEMFC). Renew. Energy.

[30] Yu M., Han Y., Li J., Wang L. (2017). One-step synthesis of sodium carboxymethyl cellulose-derived carbon aerogel/nickel oxide composites for energy storage. Chem. Eng. J..

[31] Han S., Sun Q., Zheng H., Li J., Jin C. (2016). Green and facile fabrication of carbon aerogels from cellulose-based waste newspaper for solving organic pollution. Carbohydr. Polym..

[32] Chen Y., Zhang L., Yang Y., Pang B., Xu W., Duan G., Jiang S., Zhang K. (2021). Recent progress on nanocellulose aerogels: Preparation, modification, composite fabrication, applications. Adv. Mater..

[33] Antolini E. (2020). Lignocellulose, cellulose and lignin as renewable alternative fuels for direct biomass fuel cells. ChemSusChem.

[34] Yang W., Peng Y., Zhang Y., Lu J.E., Li J., Chen S. (2018). Air cathode catalysts of microbial fuel cell by nitrogen-doped carbon aerogels. ACS Sustain. Chem. Eng..

[35] Santoro C., Serov A., Gokhale R., Rojas-Carbonell S., Stariha L., Gordon J., Artyushkova K., Atanassov P. (2017). A family of Fe-N-C oxygen reduction electrocatalysts for microbial fuel cell (MFC) application: Relationships between surface chemistry and performances. Appl. Catal. B Environ..

[36] Fauziyah M., Widiyastuti W., Setyawan H. (2020). Nitrogen-doped carbon aerogels prepared by direct pyrolysis of cellulose aerogels derived from coir fibers using an ammonia–urea system and their electrocatalytic performance toward the oxygen reduction reaction. Ind. Eng. Chem. Res..

[37] Mulik S., Sotiriou-Leventis C., Leventis N. (2008). Macroporous electrically conducting carbon networks by pyrolysis of isocyanate-cross-linked resorcinol-formaldehyde aerogels. Chem. Mater..

[38] Gu K., Kim E.J., Sharma S., Sharma P.R., Bliznakov S., Hsiao B.S., Rafailovich M.H. (2020). Mesoporous carbon aerogel with tunable porosity as the catalyst support for enhanced proton-exchange membrane fuel cell performance. Mater. Today Energy.

[39] Pandey A.P., Bhatnagar A., Shukla V., Soni P.K., Singh S., Verma S.K., Shaneeth M., Sekkar V., Srivastava O. (2020). Hydrogen storage properties of carbon aerogel synthesized by ambient pressure drying using new catalyst triethylamine. Int. J. Hydrogen Energy.

[40] Singh R., Singh M., Bhartiya S., Singh A., Kohli D., Ghosh P.C., Meenakshi S., Gupta P. (2017). Facile synthesis of highly conducting and mesoporous carbon aerogel as platinum support for PEM fuel cells. Int. J. Hydrogen Energy.

[41] Huang H., Yang S., Vajtai R., Wang X., Ajayan P.M. (2014). Pt-decorated 3D architectures built from graphene and graphitic carbon nitride nanosheets as efficient methanol oxidation catalysts. Adv. Mater..

[42] Luo J., Jang H., Sun T., Xiao L., He Z., Katsoulidis A.P., Kanatzidis M.G., Gibson J., Huang J. (2011). Compression and aggregation-resistant particles of crumpled soft sheets. ACS Nano.

[43] Zhou Y., Hu X., Guo S., Yu C., Zhong S., Liu X. (2018). Multi-functional graphene/carbon nanotube aerogels for its applications in supercapacitor and direct methanol fuel cell. Electrochim. Acta.

[44] Zhang B., Lin Z., Huang J., Cao L., Wu X., Yu X., Zhan Y., Xie F., Zhang W., Chen J. (2017). Highly active and stable non noble metal catalyst for oxygen reduction reaction. Int. J. Hydrogen Energy.

[45] Mai Z., Liu Z., Liu S., Zhang X., Cui Z., Tang Z. (2021). Atomically dispersed Co atoms in nitrogen-doped carbon aerogel for efficient and durable oxygen reduction reaction. Int. J. Hydrogen Energy.

[46] Chen Z., Liu S., Chen L., Huang J., Zheng B., Huang W., Li S., Lu Y., Fu R. (2021). A scalable molecular-templating strategy toward well-defined microporous carbon aerogels for efficient water treatment and electrocatalysis. Chem. Eng. J..

[47] Guo L., Wan K., Liu B., Wang Y., Wei G. (2021). Recent advance in the fabrication of carbon nano-fiber-based composite materials for wearable devices. Nanotechnology.

[48] Sokka A., Mooste M., Käärik M., Gudkova V., Kozlova J., Kikas A., Kisand V., Treshchalov A., Tamm A., Paiste P. (2021). Iron and cobalt containing electrospun carbon nanofiber-based cathode catalysts for anion exchange membrane fuel cell. Int. J. Hydrogen Energy.

[49] Lee Y., Motoyama Y., Tsuji K., Yoon S.-H., Mo-chida I., Nagashima H. (2012). (Z)-Selective partial hydrogenation of internal alkynes by using palladium nanoparticles supported on nitrogen-doped carbon nanofiber. Chem. Cat. Chem..

[50] Venugopal J., Ramakrishna S. (2005). Applications of polymer nanofibers in biomedicine and biotechnology. Appl. Biochem. Biotechnol..

[51] Cai T., Huang M., Huang Y., Zheng W. (2019). Enhanced performance of microbial fuel cells by electro-spinning carbon nanofibers hybrid carbon nanotubes composite anode. Int. J. Hydrogen Energy.

[52] Zhou X., Liu B., Chen Y., Guo L., Wei G. (2020). Carbon nanofiber-based three-dimensional nanomaterials for energy and environmental applications. Mater. Adv..

[53] Zhang H., Tan Y., Luo X.D., Sun C.Y., Chen N. (2019). Polarization effects of a rayon and polyacrylonitrile based graphite felt for iron-chromium redox flow batteries. ChemElectroChem.

[54] Al-Saleh M.H., Sundararaj U. (2009). A review of vapor grown carbon nanofiber/polymer conductive composites. Carbon.

[55] Zhou X., Wang Y., Gong C., Liu B., Wei G. (2020). Production, structural design, functional control, and broad applications of carbon nanofiber-based nanomaterials: A comprehensive review. Chem. Eng. J..

[56] Modi A., Singh S., Verma N. (2016). In situ nitrogen-doping of nickel nanoparticle-dispersed carbon nanofiber-based electrodes: Its positive effects on the performance of a microbial fuel cell. Electrochim. Acta.

[57] Kanninen P., Borghei M., Ruiz V., Kauppinen E., Kallio T. (2012). The effect of nafion content in a graphitized carbon nanofiber-based anode for the direct methanol fuel cell. Int. J. Hydrogen Energy.

[58] Oh Y., Kim S.-K., Peck D.-H., Jang J.-S., Kim J., Jung D.-H. (2014). Improved performance using tungsten carbide/carbon nanofiber based anode catalysts for alkaline direct ethanol fuel cells. Int. J. Hydrogen Energy.

[59] Wallnöfer E., Perchthaler M., Hacker V., Squadrito G. (2009). Optimisation of carbon nanofiber based electrodes for polymer electrolyte membrane fuel cells prepared by a sedimentation method. J. Power Sources.

[60] Chan S., Jankovic J., Susac D., Saha M.S., Tam M., Yang H., Ko F. (2018). Electrospun carbon nanofiber catalyst layers for polymer electrolyte membrane fuel cells: Structure and performance. J. Power Sources.

[61] Kvande I., Briskeby S.T., Tsypkin M., Ronning M., Sunde S., Tunold R., Chen D. (2007). On the preparation methods for carbon nanofiber-supported Pt catalysts. Top. Catal..

[62] Kvande I., Zhu J., Zhao T.J., Hammer N., Ronning M., Raaen S., Walmsley J.C., Chen D. (2010). Importance of oxygen-free edge and defect sites for the immobilization of colloidal Pt oxide particles with im-plications for the preparation of CNF-supported catalysts. J. Phys. Chem. C..

[63] Arico A.S., Antonucci P.L., Modica E., Baglio V., Kim H., Antonucci V. (2002). Effect of Pt□Ru alloy composition on high-temperature methanol electro-oxidation. Electrochim. Acta.

[64] Gasteiger H.A., Kocha S.S., Sompalli B., Wagner F.T. (2005). Activity benchmarks and requirements for Pt, Pt-alloy, and non-Pt oxygen reduction catalysts for PEMFCs. Appl. Catal. B Environ..

[65] Hung C.T., Liou Z.H., Veerakumar P., Wu P.H., Liu T.C., Liu S.B. (2016). Ordered mesoporous carbon supported bifunctional PtM (M= Ru, Fe, Mo) electrocatalysts for a fuel cell anode. Chin. J. Catal..

[66] Wang H., Yang X., Wu Q., Zhang Q., Chen H., Jing H., Wang J., Mi S.-B., Rogach A.L., Niu C. (2018). Encapsulating silica/antimony into porous electrospun carbon nanofibers with robust structure stability for high-efficiency lithium storage. ACS Nano.

[67] Zheng G.Y., Zhang Q., Cha J.J., Yang Y., Li W., Seh Z.W., Cui Y. (2013). Amphiphilic surface modification of hollow carbon nanofibers for improved cycle life of lithium sulfur batteries. Nano Lett..

[68] Sandström R., Ekspong J., Annamalai A., Sharifi T., Klechikov A., Wågberg T. (2018). Fabrication of microporous layer—Free hierarchical gas diffusion electrode as a low Pt-loading PEMFC cathode by direct growth of helical carbon nanofibers. RSC Adv..

[69] Roman J., Neri W., Derré A., Poulin P. (2019). Electrospun lignin-based twisted carbon nanofibers for potential microelectrodes applications. Carbon.

[70] Komori K., Huang J., Mizushima N., Ko S., Tatsuma T., Sakai Y. (2017). Controlled direct electron transfer kinetics of fructose dehydrogenase at cup-stacked carbon nanofibers. Phys. Chem. Chem. Phys..

[71] Merkulov V.I., Melechko A.V., Guillorn M.A., Simpson M., Lowndes D.H., Whealton J.H., Raridon R.J. (2002). Controlled alignment of carbon nanofibers in a large-scale synthesis process. Appl. Phys. Lett..

[72] Lu W.Y., He T., Xu B., He X., Adidharma H., Radosz M., Gasem K., Fan M.H. (2017). Progress in catalytic synthesis of advanced carbon nanofibers. J. Mater. Chem. A.

[73] Valdivia-Barrientos R., Pacheco-Sotelo J.O., Pacheco-Pacheco M., Ramos-Flores J.F., Cruz-Azocar A., Jimenez-Lopez M.D.L., Benitez-Read J.S., Lopez-Callejas R. (2007). Optical and electrical diagnostics of a high-frequency glow–arc discharge and its application to the synthesis of carbon nanofibers. IEEE Trans. Plasma Sci..

[74] Chung S., Ham K., Kang S., Ju H., Lee J. (2020). Enhanced corrosion tolerance and highly durable ORR activity by low Pt electrocatalyst on unique pore structured CNF in PEM fuel cell. Electrochim. Acta.

[75] Chen N., Zhang H., Luo X.D., Sun C.Y. (2020). SiO_2_-decorated graphite felt electrode by silicic acid etching for iron-chromium redox flow battery. Electrochim. Acta.

[76] Cai T., Huang Y., Huang M., Xi Y., Pang D., Zhang W. (2019). Enhancing oxygen reduction reaction of supercapacitor microbial fuel cells with electrospun carbon nano fibers composite cathode. Chem. Eng. J..

[77] Yoon K.R., Hwang C.-K., Kim S.-H., Jung J.-W., Chae J.E., Kim J., Lee K.A., Lim A., Cho S.-H., Singh J.P. (2021). Hierarchically assembled cobalt oxynitride nanorods and n-doped carbon nanofibers for efficient bifunctional oxygen electrocatalysis with exceptional regenerative efficiency. ACS Nano.

[78] Yoon K.R., Shin K., Park J., Cho S.-H., Kim C., Jung J.-W., Cheong J.Y., Byon H.R., Lee H.M., Kim I.-D. (2017). Brush-like cobalt nitride anchored carbon nanofiber membrane: Current collector-catalyst integrated cathode for long cycle Li–O_2_ batteries. ACS Nano.

[79] Jeon Y., Ji Y., Cho Y.I., Lee C., Park D.H., Shul Y.G. (2018). Oxide–carbon nanofibrous composite support for a highly active and stable polymer electrolyte membrane fuel-cell catalyst. ACS Nano..

[80] Lima R.A.C., Júnior A.J.C.P., Pocrifka L.A., Passos R.R. (2021). Investigation of nitrogen-doping influence on the electrocatalytic activity of graphene in alkaline oxygen reduction reaction. Mater. Res..

[81] Li S., Cheng C., Thomas A. (2017). Carbon-based microbial-fuel-cell electrodes: From conductive supports to active catalysts. Adv. Mater..

[82] Zhou X., Qiao J., Yang L., Zhang J. (2014). A Review of Graphene-based nanostructural materials for both catalyst supports and metal-free catalysts in pem fuel cell oxygen reduction reactions. Adv. Energy Mater..

[83] Yang L., Shui J., Du L., Shao Y., Liu J., Dai L., Hu Z. (2019). Carbon-based metal-free ORR electrocatalysts for fuel cells: Past, present, and future. Adv. Mater..

[84] Shao Y., Jiang Z., Zhang Q., Guan J. (2019). Progress in nonmetal-doped graphene electrocatalysts for the oxygen reduction reaction. ChemSusChem.

[85] Perez-Page M., Sahoo M., Holmes S.M. (2019). Single layer 2D crystals for electrochemical applications of ion exchange membranes and hydrogen evolution catalysts. Adv. Mater. Interfaces.

[86] Hu S., Lozada-Hidalgo M., Wang F., Mishchenko A., Schedin F., Nair R.R., Hill E., Boukhvalov D.W., Katsnelson M.I., Dryfe R.A.W. (2014). Proton transport through one-atom-thick crystals. Nature.

[87] Singh R.S., Gautam A., Rai V. (2019). Graphene-based bipolar plates for polymer electrolyte membrane fuel cells. Front. Mater. Sci..

[88] Navalon S., Dhakshinamoorthy A., Alvaro M., Garcia H. (2014). Carbocatalysis by graphene-based materials. Chem. Rev..

[89] Novoselov K.S., Geim A.K., Morozov S.V., Jiang D., Zhang Y., Dubonos S.V., Grigorieva I.V., Firsov A.A. (2004). Electric field effect in atomically thin carbon films. Science.

[90] Zhu Y., Murali S., Cai W., Li X., Suk J.W., Potts J.R., Ruoff R.S. (2010). Graphene and graphene oxide: Synthesis, properties, and applications. Adv. Mater..

[91] Ambrosi A., Chua C.K., Bonanni A., Pumera M. (2014). Electrochemistry of graphene and related materials. Chem. Rev..

[92] Ambrosi A., Chua C.K., Latiff N.M., Loo A.H., Wong C.H.A., Eng A.Y.S., Bonanni A., Pumera M. (2016). Graphene and its electrochemistry-an update. Chem. Soc. Rev..

[93] Farooqui U., Ahmad A., Hamid N. (2018). Graphene oxide: A promising membrane material for fuel cells. Renew. Sustain. Energy Rev..

[94] Arukula R., Vinothkannan M., Kim A.R., Yoo D.J. (2019). Cumulative effect of bimetallic alloy, conductive polymer and graphene toward electrooxidation of methanol: An efficient anode catalyst for direct methanol fuel cells. J. Alloys Compd..

[95] Qiu X., Yan X., Cen K., Sun D., Xu L., Tang Y. (2018). Achieving highly electrocatalytic performance by constructing holey reduced graphene oxide hollow nanospheres sandwiched by interior and exterior platinum nanoparticles. ACS Appl. Energy Mater..

[96] Yang H., Li S., Feng F., Ou S., Li F., Yang M., Qian K., Jin J., Ma J. (2019). Palladium nanoparticles with surface enrichment of palladium oxide species immobilized on the aniline-functionalized graphene as an advanced electrocatalyst of ethanol oxidation. ACS Sustain. Chem. Eng..

[97] Chowdhury S.R., Maiyalagan T., Bhattachraya S.K., Gayen A. (2020). Influence of phosphorus on the electrocatalytic activity of palladium nickel nanoalloy supported on N-doped reduced graphene oxide for ethanol oxidation reaction. Electrochim. Acta.

[98] Luo L., Fu C., Yang F., Li X., Jiang F., Guo Y., Zhu F., Yang L., Shen S., Zhang J. (2019). Composition-graded Cu–Pd nanospheres with Ir-doped surfaces on N-doped porous graphene for highly efficient ethanol electro-oxidation in alkaline media. ACS Catal..

[99] Yao C., Zhang Q., Su Y., Xu L., Wang H., Liu J., Hou S. (2019). Palladium nanoparticles encapsulated into hollow N-doped graphene microspheres as electrocatalyst for ethanol oxidation reaction. ACS Appl. Nano Mater..

[100] Yu K., Lin Y., Fan J., Li Q., Shi P., Xu Q., Min Y. (2019). Ternary N, S, and P-doped hollow carbon spheres derived from polyphosphazene as Pd supports for ethanol oxidation reaction. Catalysts.

[101] Peng J., He Y., Zhou C., Su S., Lai B. (2021). The carbon nanotubes-based materials and their applications for organic pollutant removal: A critical review. Chin. Chem. Lett..

[102] Huang M., Dong G., Wu C., Guan L. (2014). Preparation of highly dispersed Pt nanoparticles supported on single-walled carbon nanosheets by a microwave-assisted polyol method and their remarkable catalytic activity. Int. J. Hydrogen Energy.

[103] Lin C., Liao W., Wang W., Sun D., Cui Q., Zuo X., Yang Q., Tang H., Jin S., Li G. (2021). Self-assembled one-dimensional Co coated with N-doped carbon nanotubes for dye-sensitized solar cells with high activity and remarkable durability. CrystEngComm.

[104] Dehaghani M.Z., Yousefi F., Seidi F., Bagheri B., Mashhadzadeh A.H., Naderi G., Esmaeili A., Abida O., Habibzadeh S., Saeb M.R. (2021). Encapsulation of an anticancer drug Isatin inside a host nano-vehicle SWCNT: A molecular dynamics simulation. Sci. Rep..

[105] Yu Y., Zhao C., Tao Y., Chen X., He Y.L. (2021). Superior thermal energy storage performance of NaCl-SWCNT composite phase change materials: A molecular dynamics approach. Appl. Energy.

[106] Mu Y., Liang H., Hu J., Jiang A.L., Wan L. (2005). Controllable Pt nanoparticle deposition on carbon nanotubes as an anode catalyst for direct methanol fuel cells. J. Phys. Chem. B.

[107] Cornejo J.M., Bernabe A.G., Compan V. (2018). Bimetallic Pt-M electrocatalysts supported on single-walled carbon nanotubes for hydrogen and methanol electrooxidation in fuel cells applications. Int. J. Hydrogen Energy.

[108] Rajala T., Kronberg R., Backhouse R., Buan M.E.M., Tripathi M., Zitolo A., Luasonen K., Susi T., Jaouen F., Kallio T. (2020). A platinum nanowire catalyst on single-walled carbon nanotubes to drive hydrogen evolution. Appl. Catal. B Environ..

[109] Fernandez P.S., Castro E.B., Real S.G., Martins M.E. (2009). Electrochemical behavior of single-walled carbon nanotubes-hydrogen storage and hydrogen evolution reaction. Int. J. Hydrogen Energy.

[110] Hu R., Wu C., Hou K., Xia C., Yang J., Guan L., Li Y. (2019). Tailoring the electrocatalytic oxygen re-duction reaction pathway by tuning the electronic states of single-walled carbon nanotubes. Carbon.

[111] Wu G., Xu B.-Q. (2007). Carbon nanotube supported Pt electrodes for methanol oxidation: A comparison between multi- and single-walled carbon nanotubes. J. Power Sources.

[112] Girishkumar G., Rettker M., Underhile R., Binz D., Vinodgopal K., McGinn A.P., Kamat P. (2005). Single-wall carbon nanotube-based proton exchange membrane assembly for hydrogen fuel cells. Langmuir.

[113] Yoo J., Ju Y.-W., Jang Y.-R., Gwon O., Park S., Kim J.-M., Lee C.K., Lee S.-Y., Yeon S.-H., Kim G. (2017). One-pot surface engineering of battery electrode materials with metallic SWCNT-enriched, ivy-like conductive nanonets. J. Mater. Chem. A.

[114] Gong K., Du F., Xia Z., Durstock M., Dai L. (2009). Nitrogen-doped carbon nanotube arrays with high electrocatalytic activity for oxygen reduction. Science.

[115] Mikhayalova A.A., Tusseeva E.K., Mayorova N.A., Rychagov A.Y., Volfkovich Y.M., Krestinin A.V., Khazova Q.A. (2011). Single-walled carbon nanotubes and their composites with polyaniline, structure, catalytic and capacitive properties as applied to fuel cells and supercapacitors. Electrochim. Acta.

[116] Brennan L.J., Byrne M.T., Bari M., Gun’Ko Y.K. (2011). Carbon Nanomaterials for Dye-Sensitized Solar Cell Applications: A Bright Future. Adv. Energy Mater..

[117] Iijima S. (1991). Synthesis of carbon nanotubes. Nature.

[118] Anurag A., Niu S., Kanoun O. (2021). Effect of MWNCT dispersion parameters on the performance of electrochemical sensors. Measurement. Sensors.

[119] Vatandoust L., Habibi A., Naghshara H., Mohammad Aref S. (2021). Fabrication of ZnO-MWCNT nanocomposites sensor and investigation of its ammonia gas sensing properties at room temperature. Synth. Met..

[120] Mujahid M., Khan R.U., Mumtaz M., Sumair M., Soomro A., Ullah S. (2019). NiFe_2_O_4_ nanoparticles/MWCNTs nanohybrid as anode materials for lithium-ion battery. Ceram. Inter..

[121] Bhagwan J., Hussain S.K., Krishna B.V., Yu J.S. (2020). Facile synthesis of MnMoO_4_@MWCNT and their electrochemical performance in aqueous asymmetric supercapacitor. J. Alloys Compd..

[122] Brandão A.T., Rosoiu S., Costa R., Lazar O.A., Silva A.F., Anicai L., Pereira C.M., Enachescu M. (2021). Characterization and electrochemical studies of MWCNTs decorated with Ag nanoparticles through pulse reversed current electrodeposition using a deep eutectic solvent for energy storage applications. J. Mater. Res. Technol..

[123] Andrews R., Jaques D., Qian D.L., Rantell T. (2002). Multi-walled carbon nanotubes, synthesis and applications. Acc. Chem. Res..

[124] Zhao H., Liu X., Cao Z., Zhan Y., Shi X., Yang Y., Zhou J., Xu J. (2016). Adsorption behavior and mechanism of chloramphenicols, sulfonamides, and non-antibiotic pharmaceuticals on multi-walled carbon nanotubes. J. Hazard. Mater..

[125] Guo D.J., Cui S.K. (2021). A composite strategy to prepare high active Pt-WO_3_/MWCNT catalysts for methanol electrooxidation. J. Phys. Chem. Solids.

[126] Sun Y., Liu D., Liu W., Liu H., Zhao J., Chen P., Wang Q., Wang X., Zou Y. (2021). Fabrication of porous polyaniline/MWCNT coated Co9S8 composite for electrochemical hydrogen storage applications. J. Phys. Chem. Solids.

[127] Bhuvanendran N., Ravichandran S., Zhang W., Ma Q., Xu Q., Khotseng L., Su H. (2020). Highly efficient methanol oxidation on durable PtxIr/MWCNT catalyst for direct methanol fuel cell applications. Int. J. Hydrogen Energy.

[128] Dogan M., Selek A., Turhan O., Kiziduman B.K., Bicil Z. (2021). Different functional groups functionalized hexagonal boron nitride (h-BN) nanoparticles and multi-walled carbon nanotubes (MWCNT) for hydrogen storage. Fuel.

[129] Tian Z.Q., Jiang S.P., Liang Y.M., Shen P.K. (2006). Synthesis and characterization of platinum catalyst on multi-walled carbon nanotubes by intermittent microwave irradiation for fuel cell applications. J. Phys. Chem. B.

[130] Baker A.M., Wang L., Johnson W.B., Prasad A.K., Advani S.G. (2014). Nafion membrane reinforced with ceria coated multi-walled carbon nanotubes for improved mechanical and chemical durability in polymer electrolyte membrane fuel cells. J. Phys. Chem. C..

[131] Ghosh S., RetnaRaj C. (2010). Facile in situ synthesis of multi-walled carbon nanotube supported flower like Pt nanowire structures; An efficient electrocatalyst for fuel cell applications. J. Phys. Chem. C..

[132] Mink J.E., Hussain M.M. (2013). Sustainable design of high-performance microsized microbial fuel cell with carbon nanotube anode and air cathode. ACS Nano.

[133] Kroto H.W., Heath J.R., O Brien S.C., Curl R.F., Smalley R.E. (1985). C_60_: Buckminsterfullerene. Nature.

[134] Kausar A. (2021). Fullerene nanofiller reinforced epoxy nanocomposites—Developments, progress and challenges. Mater. Res. Innov..

[135] Baskar A.V., Benzigar M.R., Talapaneni S.N., Singh G., Karakoti A.S., Yi J., Al-Muhtaseb A.A.H., Ariga K., Ajayan P.M., Vinu A. (2021). Self-assembled fullerene nanostructures: Synthesis and applications. Adv. Fun. Mater..

[136] Hale P.D. (1986). Discrete-variation-X. Alpha. Electronic structure studies of the spherical C_60_ cluster. Prediction of ionisization potential and electronic transition energy. J. Am. Chem. Soc..

[137] Zheng T., Fan B., Zhao Y., Jin B., Fan L., Peng R. (2021). Tailored conductive fullerenes-based passivator for efficient and stable inverted perovskite solar cells. J. Colloid Interface Sci..

[138] Pontiroli D., Scaravonati S., Sidoli M., Magnani G., Fornasini L., Milanese C., Ricco M. (2019). Fullerene mixtures as negative electrodes in innovative Na-ion batteries. Chem. Phys. Lett..

[139] Uygun Z.O., Sahin C., Yilmaz M., Akay Y., Akdemir A., Sagin F. (2018). Fullerene-PAMAM(G5) composite modified impedimetric biosensors to detect fetuin-A in real blood samples. Anal. Biochem..

[140] Coro J., Suárez M., Silva L.S., Eguiluz K., Banda G. (2016). Fullerene applications in fuel cells: A review. Int. J. Hydrogen Energy.

[141] Karimi M., Aboufazeli F., Zhad H.R.L.Z., Sadegi O., Najafi E. (2013). Electroanalytical performance of Pt/Ru/Sn/W fullerene electrode for methanol oxidation in direct methanol fuel cell. J. Fuel Chem. Technol..

[142] Almeida C.V., Almagro L.E., Neto E.S.V., Coro J., Suárez M., Eguiluz K.I., Salazar-Banda G.R. (2020). Polyhydroxylated fullerenes: An efficient support for Pt electrocatalysts toward ethanol oxidation. J. Electroanal. Chem..

[143] Rambabu G., Bhat S.D. (2015). Sulfonated fullerene in SPEEK matrix and its impact on the membrane electrolyte properties in direct methanol fuel cells. Electrochim. Acta.

[144] Ahsan M.A., He T., Eid K., Abdullah A.M., Curry M.L., Du A., Santiago A.R.P., Echegoyen L., Noveron J.C. (2021). Tuning the intermolecular electron transfer of low-dimensional and metal-free BCN/C_60_ electrocatalysts via interfacial defects for efficient hydrogen and oxygen electrochemistry. J. Am. Chem. Soc..

[145] Xiao P., Buijnsters J., Zhao Y., Yu H., Xu X., Zhu Y., Tang D., Zhu J., Zhao Z. (2019). Fullerene-like WS2 supported Pd catalyst for hydrogen evolution reaction. J. Catal..

[146] Paul D.K., Karan K., Docoslis A., Giorgi J.B., Pearce J. (2013). Characteristics of self-assembled ultrathin nafion-films. Macromolcules.

[147] Rambabu G., Nagaraju N., Bhat S.D. (2016). Functionalized fullerene embedded in Nafion matrix: A modified composite membrane electrolyte for direct methanol fuel cells. Chem. Eng. J..

[148] Zeng X., Zhang J.W., Xiang P.H., Qiao J. (2018). Fabrication of graphene-fullerene hybrid by self-assembled and its applications as support materials for methanol electro oxidation reaction. Appl. Surf. Sci..

[149] Feng Y., Wang X., Huang J., Dong P., Ji J., Li J., Cao L., Feng L., Jin P., Wang C. (2020). Decorating CoNi layered double hydroxides nanosheet arrays with fullerene quantum dot anchored on Ni foam for efficient electrocatalytic water splitting and urea electrolysis. Chem. Eng. J..

